# Optimized Antimony‐Doped Tin Oxide Thin Films With Enhanced Electrical Properties for Low‐Emissivity Architectural Coatings

**DOI:** 10.1002/advs.76476

**Published:** 2026-07-09

**Authors:** Iqra Ramzan, Ivan P. Parkin, Claire J. Carmalt

**Affiliations:** ^1^ Materials Chemistry Centre Department of Chemistry University College London London UK

**Keywords:** cassiterite, chemical vapor deposition, doping, electrical resistivity and conductivity, figure of merit, glazing, materials science, optoelectronics, thin film, tin oxide

## Abstract

Antimony‐doped tin oxide (ATO) thin films were deposited by aerosol‐assisted chemical vapor deposition (AACVD) and systematically investigated to establish correlations between doping level, defect chemistry, structural properties, and optoelectronic performance. X‐ray diffraction confirmed that all films crystallized in the cassiterite structure without secondary phases, indicating successful incorporation of Sb into the SnO_2_ lattice. Moderate Sb doping significantly reduced film resistivity due to increased carrier concentration, while excessive doping resulted in increased resistivity and reduced mobility. Optical measurements revealed high visible transmittance and low haze, together with a characteristic bluish coloration at increased doping levels, attributed to free‐carrier absorption. Importantly, the films exhibited selective infrared blocking, positioning ATO as a promising candidate for solar‐control coatings. The optoelectronic performance was assessed using Haacke's figure of merit, with optimized samples demonstrating a favorable balance between electrical conductivity and optical transparency. CIE Lab* color coordinates were used to quantitatively evaluate the color appearance of the films. The controllable blue‐gray optical tint, combined with infrared heat shielding, positions AACVD‐deposited ATO films as promising candidates for low‐emissivity architectural glazing, as well as transparent electrode applications in optoelectronic devices.

## Graphical abstract

Antimony‐doped tin oxide (ATO) thin films were deposited by aerosol‐assisted chemical vapor deposition (AACVD) and systematically investigated to establish correlations between Sb doping level, structural properties and optoelectronic performance. Optimized films exhibited low sheet resistance, high carrier concentrations, and high visible transparency comparable to sputtered ATO films. The results demonstrated AACVD as a scalable route for high‐performance transparent conducting oxide coatings with potential for low‐emissivity glazing applications.

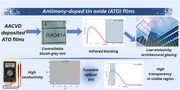

## 1 Introduction

Transparent conducting oxides (TCOs) are essential materials in modern optoelectronic applications, including photovoltaics, display technologies, and smart windows, owing to their unique combination of high optical transparency and electrical conductivity [1, 2]. The commercial value of producing sustainable and efficient TCOs is significant. For a material to be considered as a TCO, it must exhibit a wide bandgap (≥ 3.1 eV), low electrical resistivity (≤10^−3^), a carrier concentration below 10^22^ cm^−3^, and high electron mobility [1]. The electrical resistivity depends on the density of charge carriers and their mobility [1, 3].

Commercial demands for continuous performance improvements, combined with the limited supply of indium, the key component of the widely used indium‐tin oxide (ITO), have driven significant research into finding alternative TCO materials [1, 2, 4]. Tin oxide (SnO_2_) and zinc oxide (ZnO), both derived from inexpensive and abundant raw materials, have gained particular attention [5, 6, 7]. These metal oxides are typically intrinsic n‐type, direct wide‐bandgap semiconductors, with properties that can be tuned through selective doping. SnO_2_‐based films are often preferred due to their resistance to chemical attack, thermal degradation, and mechanical durability, making them ideal for thin film applications in commercial products [6, 7, 8, 9].

Thin films based on tin(IV) oxide have a wide fundamental bandgap (>3.6 eV) and strong stability, making them a good and cost‐effective alternative to the more expensive indium‐tin oxide (ITO) films [10, 11, 12, 13]. Improving electrical n‐type conductivity can be achieved by adding intrinsic defects, like oxygen vacancies, or by using extrinsic defects through aliovalent dopants, such as higher‐valency cations [8, 9, 10, 11]. For nominally undoped SnO_2_, the resistivity is typically limited to the range of 10^2^–10^3^ Ω cm [14, 15, 16]. Dopants such as fluorine and antimony have widely been reported to enhance the n‐type conductivity of SnO_2_, with antimony doping also resulting in blue‐colored films [8, 9]. However, excessive doping is not desirable as it can negatively affect conductivity by degrading the film structure, which leads to a reduction in the mobility of free charge carriers [17, 18, 19, 20].

In this work, the impact of Sb‐doping concentration on the structural, optical and electrical properties of SnO_2_ is systematically investigated. Particular attention is given to the resulting functional properties of the films, including infrared shielding, optical appearance, and electrical conductivity. The Sb‐doped SnO_2_ (ATO) films exhibit a blue coloration originating from free‐carrier absorption associated with high electron concentrations, indicating degenerate doping. Such optical behavior is technologically attractive because it enables selective infrared absorption while preserving visible transparency. The bluish tint combined with strong infrared blocking makes these films promising for architectural and automotive glazing applications, where reduction of solar heat gain is required without compromising visible light transmission. Furthermore, the controllable optical tint and electrical conductivity make ATO films attractive for decorative coatings, electrochromic systems and other optoelectronic devices where both functionality and appearance are important. High visible transmittance combined with selective near‐infrared absorption therefore positions ATO as a promising candidate for energy‐efficient windows and optoelectronic applications.

In this study, SnCl_4_ and SbCl_5_ are used in combination as precursors to deposit ATO films by AACVD. While SnCl_4_ is widely used as an industrial precursor for the deposition of fluorine‐doped tin oxide (FTO) coatings via atmospheric pressure chemical vapor deposition (APCVD), its use for the scalable AACVD deposition of ATO films has not previously been explored. The use of SbCl_5_ precursor enabled effective control of Sb^5+^ incorporation without contributions from Sb^3+^ species, providing improved control over optoelectronic properties. Previously, the use of antimony(III) ethoxide in combination with butyltin trichloride has been reported to produce ATO films containing mixed Sb^5+^ and Sb^3+^ oxidation states, which has been found to have a detrimental effect on the structural and functional properties of the films [21].

To optimize optoelectronic properties, SnO_2_ was doped with varying concentrations of antimony. The resulting doped SnO_2_ structure incorporated antimony in predominantely +5 state, contributing to the bluish coloration of the films. We demonstrate that films properties can be precisely controlled by varying dopant concentration and misting time. Our optimized ATO thin films exhibited significantly improved electrical and structural properties compared to the undoped SnO_2_.

Various techniques are used for depositing SnO_2_ thin films, including photochemical methods, sol‐gel processing, chemical vapor deposition (CVD), spin coating, dip coating, and spray pyrolysis. Among them, aerosol‐assisted chemical vapor deposition (AACVD) stands out as a superior technique due to its simplicity, scalability, and cost effectiveness. AACVD not only eliminates the requirement for highly volatile precursors, expanding the range of suitable materials, but also enables the deposition of uniform, and adhesive films with excellent control over dopant incorporation [22]. Furthermore, AACVD offers high growth rates and the capability to produce large‐area thin films, making it highly suitable for scalable deposition of functional coatings with industrial relevance [18].

## 2 Experimental Details

All chemicals were used as received without further purification: tin(IV) chloride (SnCl_4_; 99.995%, Sigma–Aldrich), antimony(V) chloride (SbCl_5_; 99.99%, Sigma–Aldrich), methanol (MeOH; 99.99% Fischer), and ethyl acetate (99.5%, Fischer Scientific). Compressed air was used as supplied by BOC. The glass substrate was 3.2 mm thick, plain float glass with a 50 nm thick SiO_2_ barrier layer, supplied by Pilkington NSG.

### 2.1 Synthesis

Depositions were carried out in a flat‐bed, cold‐walled tubular AACVD reactor, as described previously for related systems. A graphite block fitted with a Whatman cartridge heater and monitored by a Pt‐Rh thermocouple was positioned in the lower half of the reactor to control substrate temperature. Pilkington silica‐coated barrier glass (float glass with a 50 nm SiO_2_ layer on one side) was used as the substrate. The films were deposited on the coated side to suppress ion diffusion from the glass into the growing film. Substrates (15 cm × 4 cm) were cleaned with soapy water and isopropanol, then dried in an oven at 70°C. To maintain laminar aerosol flow and improve film uniformity, a second piece of barrier glass was suspended 5 mm above the substrate.

The precursor solution was prepared by dissolving 0.05 mL of SnCl_4_ and dopant amounts of SbCl_5_ in 14 mL of solvents (7 mL ethyl acetate + 7 mL methanol) in a glass bubbler. The Sb precursor concentration was varied by adding controlled volumes of SbCl_5_ to the SnCl_4_ solution. Specifically, 0.32, 0.97, 1.94, and 3.24 µL of SbCl_5_ were used to obtain ATO‐1, ATO‐2 (and ATO‐5), ATO‐3, and ATO‐4, respectively, corresponding to nominal Sb doping levels of 1, 3, 6, and 10 mol% relative to SnCl_4_ (Table 1). Aerosols were generated using a piezoelectric device and delivered into the reactor by compressed air at 1 L min^−1^. The substrate was heated to the deposition temperature (590°C) prior to deposition and cooled to room temperature before taking out the substrate for further analysis.

### 2.2 Analysis

Grazing‐incident x‐ray diffraction (GIXRD) was carried out on a Panalytical Empyrean diffractometer using Cu Kα radiation (λ = 1.5406 Å) at 40 kV and 40 mA. The incident beam angle was fixed at 1°, and diffraction patterns were collected over 10°–70° in 0.05° steps at a rate of 0.5° s^−1^. Peak positions were assigned by comparison with reference data from the Inorganic Crystal Structure Database (ICSD). The recorded patterns were analyzed for crystallinity and preferred orientation and plotted using OriginPro.

The diffraction peaks were analyzed using a Gaussian peak profile. The peak positions (2θ) and the full width at half maximum (FWHM) values were obtained from the fitted peaks. The lattice parameters (*a* and *c*) were calculated from several diffraction peaks by applying Bragg's law together with the calculations for a tetragonal crystal structure. The uncertainties were estimated from the standard deviation of the values calculated from different reflections, giving an estimated uncertainty of ±0.001 Å. The unit cell volume was calculated using *V*  = *a* ^2^ *c*, and the related uncertainty (±0.04 Å^3^) was determined using standard error propagation based on the lattice parameters.

The average crystallite size was estimated using the Scherrer equation, using the FWHM values obtained from the Gaussian‐fitted peaks. By considering uncertainties in the determination of peak width, the crystallite sizes were estimated to have an uncertainty of approximately ±10%.

X‐ray photoelectron spectroscopy (XPS) was performed on a Thermo Scientific spectrometer with a monochromatic Al Kα source. High‐resolution scans of the Sn 3d, Sb 3d, O 1s, and C 1s regions were acquired using a pass energy of 50 eV and a spot size of 400 µm. Spectra were fitted in Avantage software, with binding energies calibrated against the adventitious C 1s peak at 284.5 eV. The final modeled spectra were plotted using OriginPro. The core‐level spectra were fitted using a mixed Gaussian–Lorentzian peak shape, and the fitting procedure was carried out using the Powell optimization algorithm. A Smart background subtraction was applied before performing the fitting. The uncertainties in the XPS atomic percentages were estimated by repeating the peak fitting for spectra collected from three different areas of each sample.

Film morphology was studied by scanning electron microscopy (SEM) in top‐down mode using a JEOL JSM‐7600 field‐emission microscope operated at 5–15 kV. Prior to imaging, samples were carbon coated to improve contrast and ensure accurate EDS measurements. Energy‐dispersive x‐ray spectroscopy (EDS) was used to locate the presence of different elements present in the films with much focus on residual chlorine and Sb as dopant.

The film thicknesses were determined using the Bruker Dektak XT instrument, operating at room temperature. Film thickness was measured on a 1 × 1 cm area of each sample, and the electrical properties were measured on adjacent 1 cm × 1 cm areas to ensure accurate and representative electrical property data. From each 15 cm substrate, the first 2 cm section was used for thickness and electrical measurements, while the region between 3 and 6 cm was used for optical measurements to maintain consistency in the data.

**TABLE 1:** Precursor solution misting time, crystallite size, lattice parameters, antimony and chlorine content, O/Sn ratio (from XPS data), and the carrier mean free path of undoped and Sb‐doped SnO_2_ thin films prepared by AACVD.

Film	Misting time (min)	Crystallite diameter (nm)	*a*/ Å (±0.001Å)	*c*/Å(±0.001Å)	Cl (at.%)	Sb (at.%)	O/Sn	Mean free path (nm)
**Undoped SnO_2_**	05	28.1 ± 1.0	4.742	3.243	0.6 ± 0.02	—	1.7 ± 0.10	3.0
**ATO‐1**	05	29.5 ± 1.3	4.750	3.197	0.6 ± 0.02	1.3 ± 0.01	1.6 ± 0.09	2.5
**ATO‐2**	05	30.6 ± 1.5	4.745	3.196	0.8 ± 0.03	4.7 ± 0.02	1.7 ± 0.12	2.3
**ATO‐3**	05	30.3 ± 1.7	4.754	3.199	0.7 ± 0.01	6.5 ± 0.03	1.8 ± 0.15	0.8
**ATO‐4**	05	36.9 ± 0.9	4.749	3.195	0.7 ± 0.04	8.1 ± 0.02	1.5 ± 0.10	0.3
**ATO‐5**	10	31.7 ± 2.0	4.748	3.199	0.6 ± 0.04	5.2 ± 0.01	1.9 ± 0.10	2.7

Electrical properties were measured by Hall effect measurements using a four‐point probe in Van der Pauw geometry on an Ecopia HMS‐3000 setup. Recordings were performed on 1 × 1 cm^2^ samples with an applied current of 1 mA under a calibrated magnetic field of 0.58T. It provided the values of resistivity, mobility, and carrier concentration. Each sample was reproduced three times to ensure the reproducibility and reliability of the results. The error bars presented in Table 2 represent the standard deviations calculated from these three repeats.

**TABLE 2:** Film thickness, electrical properties (including calculated sheet resistance and figure of merit), optical bandgap, transmittance at 550 nm and average transmittance in the visible (380–750 nm), near‐infrared (750–1400 nm), and short‐wave infrared (1400–2500 nm) ranges for SnO_2_ films and the TEC‐15 reference.

Film	Film Thickness (± 50 nm)	Resistivity (Ω cm)	Mobility (cm^2^ V^−1^ s^−1^)	Carrier Conc. (cm^−3^)	Sheet Resistance (Ω □^−1^)	F.O.M. (Ω^−1^)	Bandgap (eV)	T_λ550_ (%)	T_λ380‐750_ (%)	T_λ750‐1400_ (%)	T_λ1400‐2500_ (%)	Haze (%)380–750 nm
Undoped SnO_2_	485	8.9 (±0.5) × 10^−4^	21.5	3.3 × 10^20^	18.4	4.6 × 10^−3^	3.93 (±0.03)	78.1 (±0.6)	78.3	82.3	72.0	2.7
ATO‐1	619	9.8 (±0.5) × 10^−4^	17.0	3.8 × 10^20^	15.8	0.8 × 10^−3^	3.99 (±0.03)	64.4 (±0.5)	63.9	35.1	1.2	6.0
ATO‐2	686	5.3 (±0.6) × 10^−4^	11.1	1.0 × 10^21^	7.7	2.4 × 10^−3^	4.0 (±0.01)	67.0 (±0.5)	64.7	37.7	1.3	5.5
ATO‐3	743	1.3 (±0.3) × 10^−3^	3.7	1.3 × 10^21^	17.5	0.9 × 10^−3^	4.01 (±0.01)	66.0 (±0.4)	64.1	37.5	2.6	8.5
ATO‐4	879	3.9 (±0.7) × 10^−3^	1.6	1.1 × 10^21^	44.4	0.01 × 10^−3^	4.02 (±0.01)	46.7 (±0.3)	44.4	15.0	0.14	6.0
ATO‐5	920	2.0 (±0.4) × 10^−4^	8.5	3.6 × 10^21^	2.2	1.5 × 10^−3^	4.06 (±0.02)	56.5 (±0.4)	55.4	44.3	15.4	21.0
TEC 15	340	4.5 × 10^−4^	34.0	4.0 × 10^20^	13.2	13.7	3.97	84.3	83.4	74.3	25.5	—

Optical properties were measured at room temperature using a Shimadzu UV–vis–NIR 3600i Plus spectrometer in the 200–2500 nm range. Bandgaps were derived from transmittance spectra (converted to absorbance) using Tauc plot method in OriginPro.

CIE L*a*b* color coordinates were calculated from the measured transmittance data under the CIE D65 illuminant using standard colorimetric calculations. The spectral power distribution of the CIE standard illuminant D65 and the CIE 1964 color‐matching functions (10° standard observer) were obtained from the International Commission on Illumination (CIE) database [23, 24]. Tristimulus values (X, Y, Z) were obtained by integrating the transmittance spectra with the CIE color matching functions and subsequently converted into L*, a*, and b* values using the standard nonlinear CIE transformation. The resulting color coordinates were plotted in OriginPro to visualize color evolution upon Sb‐doping.

Finally, static water contact angle measurements were carried out using a Krüss DSAE droplet shape analyzer employing the sessile drop method. Ultrapure water droplets with a volume of 5 µL were used under ambient conditions. The contact angles were determined from the recorded droplet images using the instrument's image analysis software.

## 3 Results and Discussion

Undoped and Sb‐doped SnO_2_ (ATO) thin films were deposited by AACVD on barrier glass substrates using SnCl_4_ and SbCl_5_ precursors dissolved in a mixed‐solvent system (7 mL ethyl acetate + 7 mL methanol). The Sb doping level was tuned across the series (**ATO‐1** to **ATO‐4**) to yield increasing Sb contents, while maintaining the same deposition temperature and baseline misting duration of 05 min.

To evaluate the effect of deposition time or thickness independently of solution chemistry, **ATO‐5** was prepared using the same precursor chemistry as **ATO‐2** but with an extended misting duration of 10 min (Table 1). It enabled the assessment of how additional precursor delivery can influence microstructure and functional properties.

Residual chlorine was detected in the films by XPS, which is expected when using chlorine based precusrsors such as SnCl_4_ and SbCl_5_. No significant variation in chlorine content was observed with increasing Sb concentration and was therefore treated as a secondary factor affecting the electrical response.

All films showed excellent substrate coverage and strong adhesion (verified by the Scotch tape test), and remained stable in air with no measurable change in electrical properties over 14‐months period. The Sb‐doped SnO_2_ films appeared blue in coloration, with the tint becoming more pronounced at higher dopant levels. The strong near‐IR absorption can make the films an attractive choice for solar‐control or heat‐shielding glazing while retaining visible transparency.

A blue‐gray appearance is an established aesthetic in architectural glazing. For example, the triple‐silver low‐E product “Solarban 70 Optiblue” is specified to provide solar‐control performance while maintaining a steel blue‐gray visual tone [25]. It is also reported that Ag‐based low‐E coatings can be optically engineered for controlled tint, reinforcing that a slight blue or gray tone is compatible with high‐performance low‐E glazing applications [26].

**FIGURE 1:**
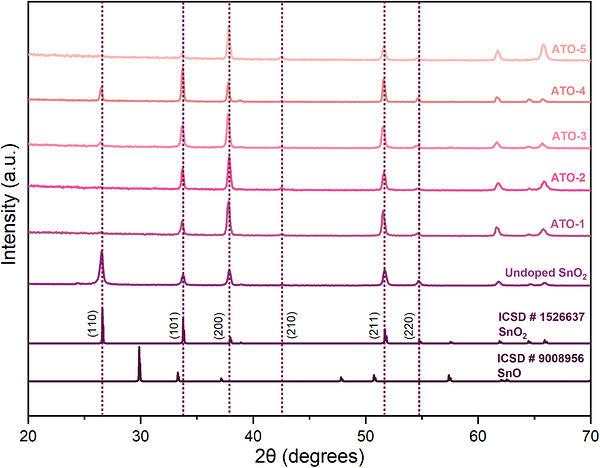
Collated XRD diffractograms of SnO_2_ and ATO films.

The x‐ray diffraction (XRD) patterns of the as‐deposited films (Figure 1) confirmed that the samples were phase‐pure and crystallized in the cassiterite structure of SnO_2_, characterized by a tetragonal rutile‐type lattice (space group P4_2_/mnm). No secondary phases or impurity peaks were detected, indicating that high‐purity SnO_2_ films were obtained under the applied deposition conditions. The films exhibited a polycrystalline nature, with distinct diffraction peaks observed at 26.5°, 33.7°, 37.9°, 42.6°, 51.7°, 54.7°, 61.8°, 64.6°, and 65.9°, corresponding to the (110), (101), (200), (210), (211), (220), (310), (112), and (301) planes, respectively. These peaks were indexed to ICSD #1526637 (space group P4_2_/mnm, a = b = 4.736 Å, c = 3.201 Å, V = 71.812 Å^3^), as shown in Figure 1.

The crystallite sizes (D) were determined through the Scherrer formula [27], as shown in Equation 1:

(1) $$D=\frac{k\lambda }{\beta cos\theta_{B}}\;$$

The calculated unit cell parameters and crystallite sizes are listed in Table 1. No antimony oxide peaks were detected even at the highest dopant concentrations, suggesting that a solid solution (or a significant amorphous component) was successfully formed. The crystallite size of the ATO film was larger compared to the undoped film. This indicates improved crystal growth and nucleation, with reduced lattice distortion, which is beneficial for producing films with optimized carrier transport properties [28]. A similar broadening of XRD peaks with increasing antimony doping has also been reported by Krishnakumar et al. [29]. The undoped SnO_2_ film exhibited a crystallite size of 28.1 nm, which increased progressively upon Sb addition, reaching a maximum of 36.9 nm for ATO‐4. This trend suggests that Sb incorporation improved crystallinity and grain growth [30, 31].

By increasing the misting time of the precursor solution from 05 min (**ATO‐2**) to 10 min (**ATO‐5**), the crystallite size (shown in Table 1) increased from 30.6 to 31.7 nm. The availability of precursor species for longer time likely led to a higher rate of nucleation and growth and allowed existing crystallites to grow larger over the extended deposition period. This suggests that the misting time is a key parameter for controlling the final crystallite size and film thickness.

The variation of the lattice parameters with Sb incorporation was analyzed to assess the structural impact of doping. As shown in Table 1, the *c* value decreases from 3.243 Å in the undoped SnO_2_ film to ∼3.195 Å in **ATO‐4**. The contraction along the *c*‐axis upon Sb addition is consistent with substitutional incorporation of Sb^5+^ at Sn^4+^ lattice sites, given the comparable ionic radii of Sb^5+^ (∼0.60 Å) and Sn^4+^ (∼0.69 Å) in sixfold coordination, which allows incorporation without lattice expansion. In contrast, Sb^3+^ possesses a significantly larger ionic radius (∼0.76 Å), and its substitution is typically associated with lattice expansion. The observed decrease in the *c* parameter therefore further supports the incorporation of Sb predominantly in the +5 oxidation state, in agreement with the XPS analysis.

In thin films, growth can be anisotropic, meaning that certain crystal planes may grow faster or be more energetically favorable to align in a specific direction. To quantify this preferential orientation, texture coefficient (TC) values were calculated using Equation 2 [32]. A TC value greater than 1 for a particular (hkl) plane indicates a strong preferred orientation along that direction.

(2)

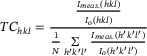

where I_meas._ is the measured intensity of individual (hkl) plane reflection, I_o_ is the theoretical intensity obtained from the ICSD data, and N is the total number of reflections included in calculation.

The electrical properties of ATO films can be influenced by their crystallographic orientation due to the intrinsic anisotropy of the cassiterite (rutile) structure. In rutile SnO_2_, the SnO_6_ octahedra share edges along the [001] direction (the *c*‐axis), forming linear chains that may facilitate charge transport due to orbital overlap along this axis. XRD analysis in this study revealed a preferred orientation along the (200) plane. In the tetragonal system, the [100] direction is normal to the (200) planes. Therefore, for (200)‐textured growth, the [001] direction (the *c*‐axis) lies within the plane of the film. All the films showed preferred orientation in (200) plane. The (200) plane is a low‐density plane believed to be associated with the presence of both oxygen vacancies and/or dopants. This specific plane is predicted to have fewer deep‐lying trap levels at grain boundary surfaces, which may contribute to a higher degree of conductivity across the film [33, 34, 35]. This geometrical alignment may be favorable for in‐plane charge transport, as it aligns the potentially more conductive crystallographic direction with the direction of electrical measurement. The (210) plane also showed TC values above 1 for all the samples. The TC values of (200) and (210) planes are shown in Figure S1. However, as the films are polycrystalline, electrical transport is not governed solely by crystallographic orientation but is also influenced by microstructural factors such as grain boundaries, defect states and dopant distribution.

**FIGURE 2:**
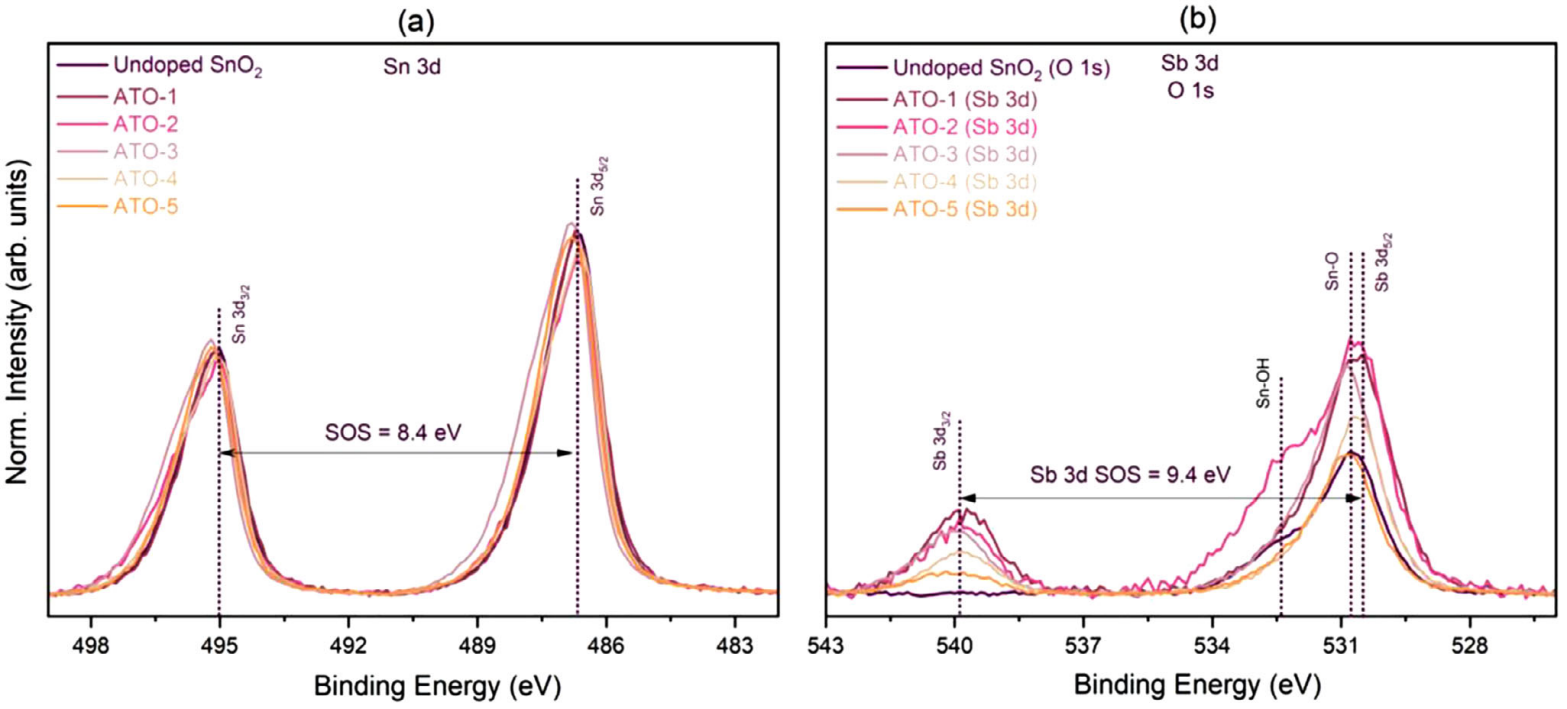
XPS spectra of (a) Sn 3d (b) Sb 3d.

X‐ray photoelectron spectroscopy (XPS) was used to analyse the surface chemical composition, electronic states, and elemental quantification of the tin oxide films. The Sn 3d spectra of all samples are presented in Figure 2a. The binding energy of Sn 3d_5/2_ was observed in the range of 486.5–486.9 eV, consistent with the reported value for stoichiometric SnO_2_ (486.6 eV). The spin‐orbit splitting between Sn 3d_5/2_ and Sn 3d_3/2_ was 8.4 eV [36, 37]. No additional contributions associated with Sn(II), typically detected at slightly lower binding energy (∼486.0 eV), were identified [29].

Upon Sb doping, the Sn 3d core‐level peaks showed a positive shift of ∼0.1–0.2 eV relative to undoped SnO_2_. This shift can be attributed to the upward movement of the Fermi level caused by donor electrons introduced through Sb^5+^ substitution on Sn^4+^ lattice sites [38, 39], which increases the carrier concentration and reflects the enhanced n‐type conductivity of the films. The spin‐orbit splitting of the Sn 3d peaks remained unchanged, confirming that the chemical environment of tin is preserved.

The Sb 3d peak is superposed with that of O 1s, thus the deconvolution of the peaks was not straightforward. The Sb 3d and O 1s spectra exhibited complex features due to the overlapping of O1s and Sb 3d_5/2_ photoemission lines at around 531 eV [40]. Hence, spectral deconvolution was performed while accounting for spin‐orbit coupling, with the Sb 3d_3/2_ and Sb 3d_5/2_ area ratio set to 2:3 and the doublet splitting energy fixed at 9.3 eV. This method allowed for the independent quantification of the contributions from the Sb 3d and O 1s peaks. Moreover, the less intense 3d_3/2_ peak is an indicative of the presence of Sb in the films.

The Sb 3d_5/2_ and 3d_3/2_ peaks for Sb^3+^ are typically reported around 528.9 and 538.7 eV, respectively [41, 42]. However, Sb(V) peaks appear at slightly higher binding energies [43, 44, 45]. In the recorded Sb 3d spectra, no features attributable to Sb^3+^ were detected, and the observed doublet was therefore assigned to Sb^5+^. The contributions from O1s were deconvoluted into two components to determine the bonding states of different oxygens present in the samples. The first peak centered at approximately 530.7 eV was assigned to lattice oxygen corresponding to Sb─O bonds. Similar O 1s signals at approximately same binding energies have been previously reported and linked to the metal‐oxygen bond [41, 42, 46, 47, 48, 49]. However, the higher binding energy peak at around 532.3 eV was attributed to surface hydroxyl group (─OH) [36, 50]

A computational investigation using hybrid density functional theory (DFT) was performed by Sapna D. Ponja et al. to explore the role of intrinsic and Sb‐related defects in SnO_2_ [21]. In their study, ATO films deposited by AACVD were found to contain antimony in both Sb^5+^ and Sb^3+^ oxidation states, which was attributed to the use of an Sb(III) precursor (SbCl_3_). In contrast, the use of an Sb(V) precursor in the present work resulted exclusively in the incorporation of Sb^5+^ species within the films, thereby yielding improved electronic properties compared with previously reported AACVD‐derived ATO films [21, 51, 52].

**FIGURE 3:**
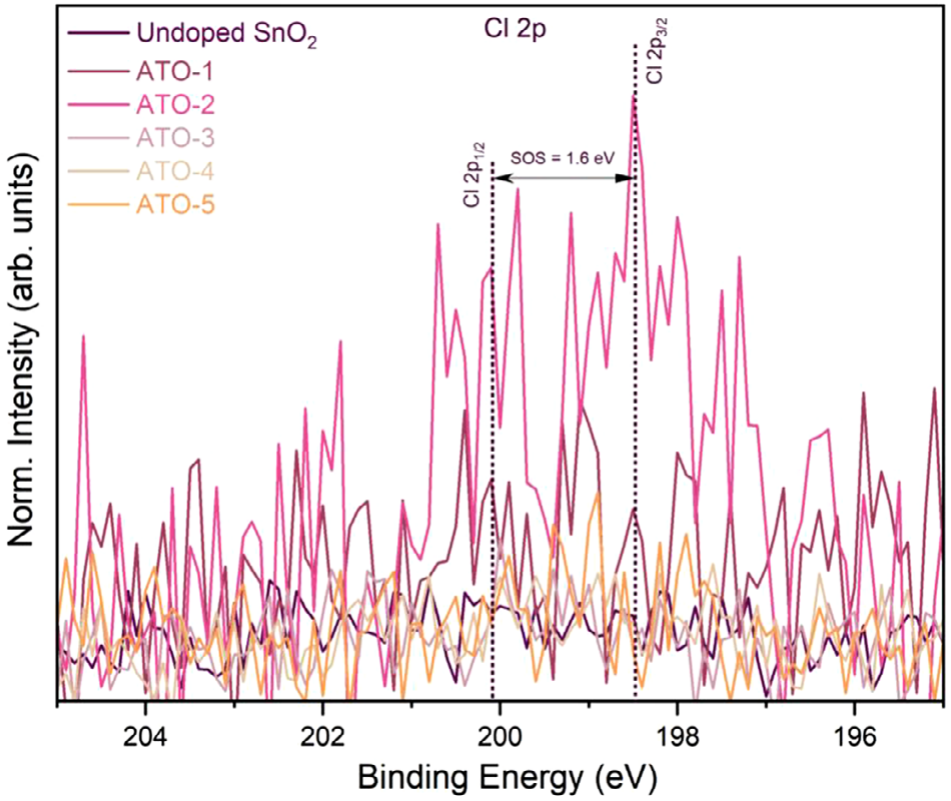
XPS spectra of Cl 2p.

The Cl 2p spectra (Figure 3) were analyzed to determine the chemical state of residual chlorine in the films. A weak and noisy doublet corresponding to the Cl 2p_3/2_ and Cl 2p_1/2_ components was observed, with the first peak located at approximately 198.7 eV. This position is consistent with chlorine in a chloride‐like environment, likely present as an impurity at grain boundaries or substituting for oxygen within the SnO_2_ lattice [36]. The spin‐orbit splitting of the Cl 2p doublet was determined to be 1.6 eV. Due to the low concentration of chlorine (<1 at.%), the Cl 2p signal was relatively weak. However, peak fitting was performed to quantify it, and a representative deconvoluted Cl 2p spectrum of **ATO‐2** is provided in Figure S2.

**FIGURE 4:**
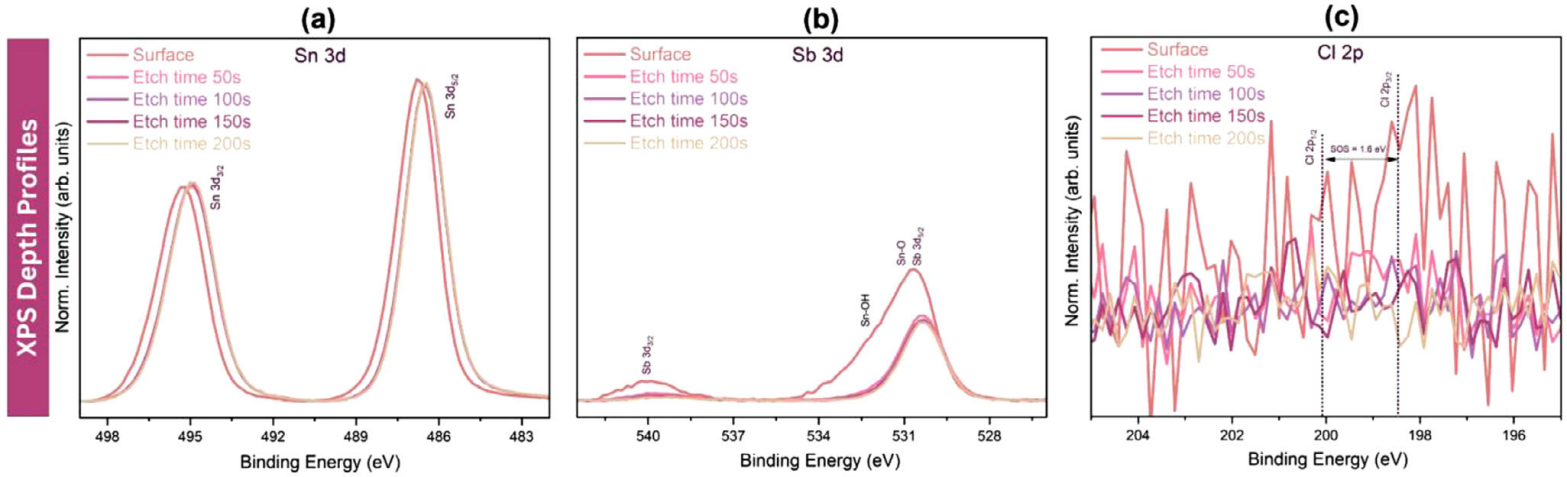
XPS depth profiling of ATO‐2 (a) Sn 3d (b) Sb 3d (c) Cl 2p.

Depth profiling of Sn 3d, Sb 3d, and Cl 2p for sample ATO‐2 was carried out up to four sputtering levels, with each level corresponding to 50 s etching (Figure 4). The chlorine concentration was highest at the surface (0.8 at.%) and gradually decreased with depth, measuring 0.7 at.% at the first etch level, 0.7 at.% at the second and third levels, and 0.5 at.% at the fourth level. This trend indicates a slight surface enrichment of chlorine with a relatively uniform distribution throughout the bulk of the film. The Sb concentration exhibited a similar depth profile, with 4.7 at.% at the surface gradually decreasing to 4.1 at.% at the fourth etch level. This suggests that the dopant was not predominantly surface segregated but rather quite uniformly distributed throughout the film.

**FIGURE 5:**
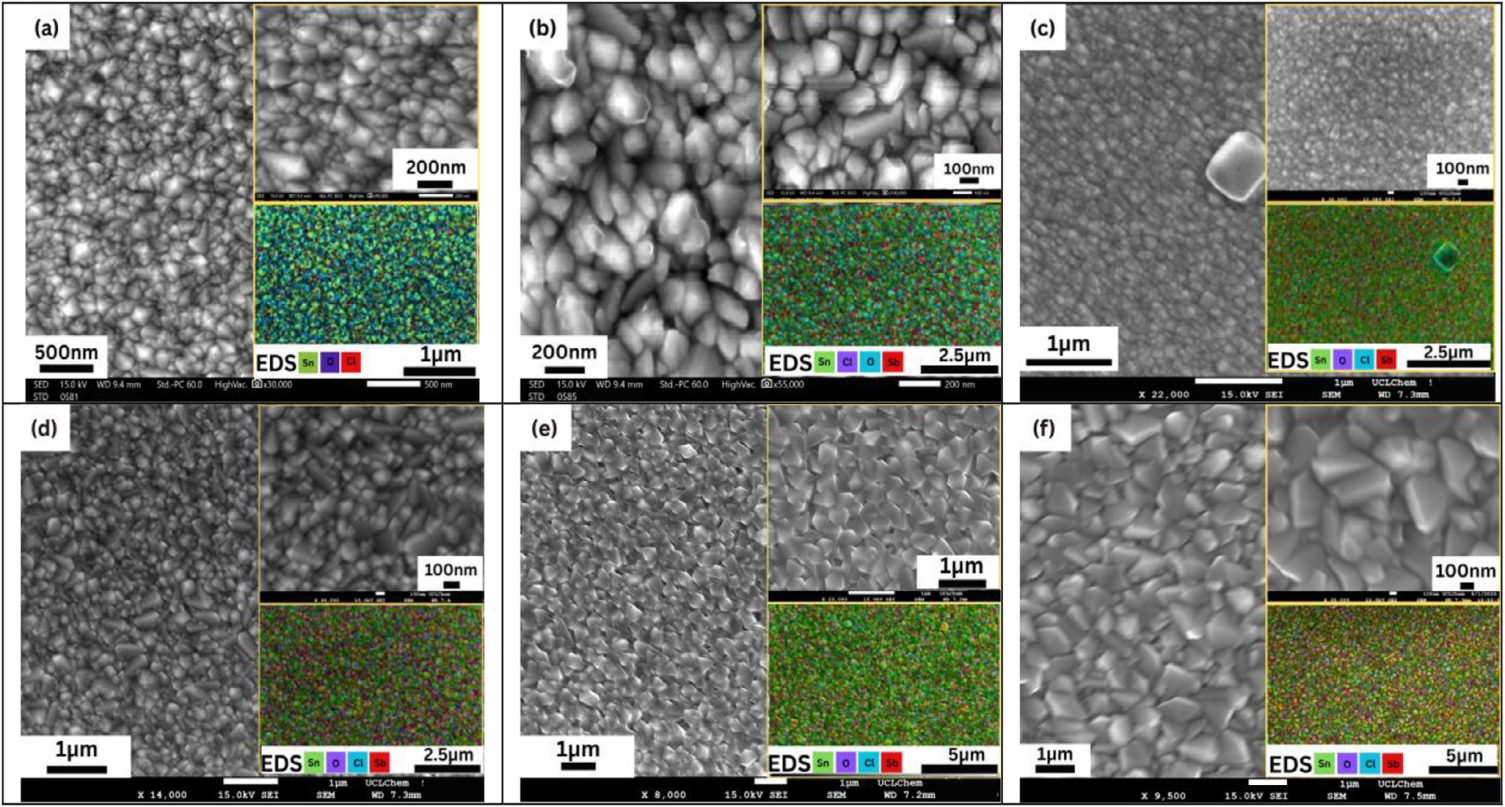
SEM and EDS images of (a) Undoped SnO_2_ (b) ATO‐1 (c) ATO‐2 (d) ATO‐3 (e) ATO‐4 (f) ATO‐5.

Scanning electron microscopy (SEM) was used to determine the surface morphology of the films. The SEM images, as shown in Figure 5, revealed noticeable changes in surface features with the changes in dopant concentration and misting time. The undoped SnO_2_ film showed compact surface morphology consisting of closely packed grains, which is characteristic of pure SnO_2_ [14, 15, 16, 53, 54]. The grain boundaries were quite defined, and no evidence of cracking or voids was observed. This showed homogeneous nucleation under the applied AACVD conditions.

Upon introduction of Sb dopant at low to moderate concentrations (**ATO‐1** to **ATO‐3**), the films noticeable increase in grain size and retained dense and continuous morphology. The grains appeared more faceted, suggesting enhanced surface diffusion and crystal growth promoted by Sb incorporation. The observed changes in grain growth agreed well with the changes in crystallite size observed under XRD. The improved carrier transport properties can be attributed to improved grain growth and reduced grain boundaries. Decrease in mobility can be attributed to ionized impurity scattering [55]. Such highly textured surface morphologies can be advantageous for TCO applications, such as solar cells, which benefit from improved light trapping and minimize reflection losses [17, 18, 35, 56, 57, 58, 59].

At higher Sb concentration (**ATO‐4**), the surface became more heterogeneous with smaller and irregularly shaped grains. The presence of poorly connected grains suggested that excessive Sb incorporation disrupted normal crystal growth. The effect of extended misting time is evident when comparing **ATO‐2** and **ATO‐5**. Despite similar solution chemistry, **ATO‐5** showed larger grains. The prolonged supply of precursor allowed sustained grain growth, resulting in improved inter‐grain connectivity. Importantly, SEM confirmed that thickness‐related improvements in conductivity arise from microstructural evolution rather than increased defect density, which would otherwise compromise optical performance.

Energy‐dispersive x‐ray spectroscopy (EDS) mapping performed alongside SEM indicated a relatively uniform distribution of Sb across the film surface. This observation is consistent with the XRD and XPS results, suggesting that Sb is relatively uniformly distributed throughout the film. Such relatively uniform dopant distribution is beneficial for achieving consistent electrical properties across large‐area films and highlights the effectiveness of AACVD for controlled dopant incorporation.

**FIGURE 6:**
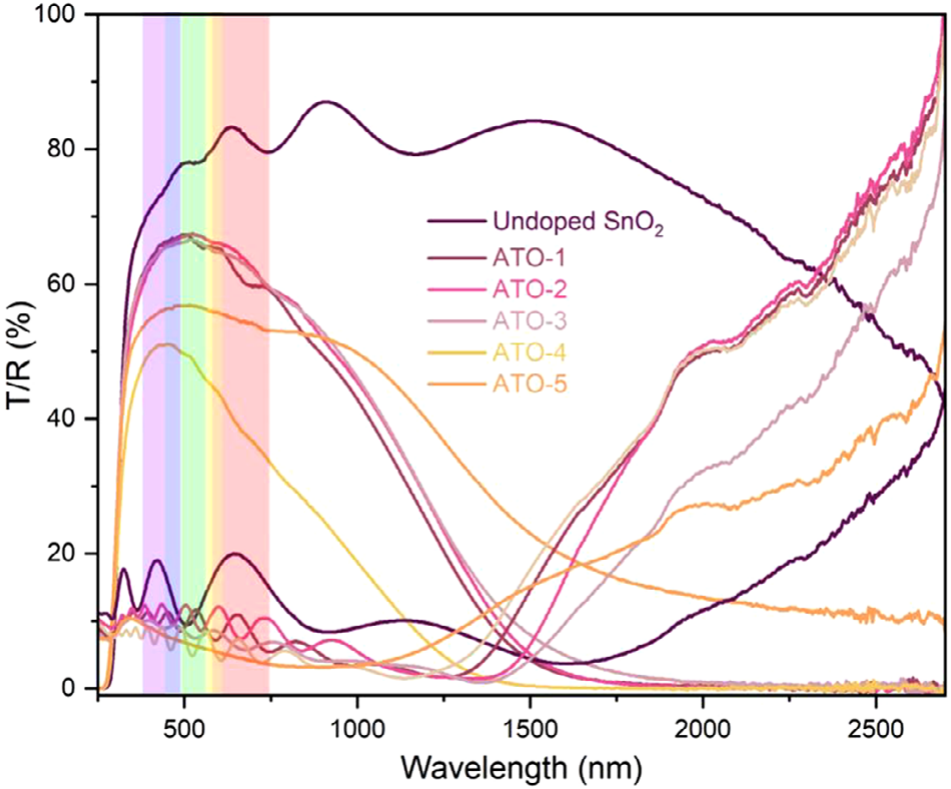
Transmittance (T) and reflectance (R) spectra of undoped SnO_2_ and ATO samples.

The optical properties of the SnO_2_ films were determined using UV–vis spectroscopy. The transmittance and reflectance spectra of all the samples are shown in Figure 6. The optical transmittance of undoped and ATO films at 550 nm and average values across the visible (380–750 nm), near‐infrared (750–1400 nm), and short‐wave (1400–2500 nm) regions are shown in Table 2. The transmittance and reflectance spectra of commercial FTO (TEC‐15, NSG Pilkington), and uncoated float glass are shown in Figure S3. TEC‐15 showed a transmittance of 84.3% at 550 nm, as shown in Table 2.

The doped films exhibited lower transmittance compared to the undoped SnO_2_ film (Figure 6). The transmittance T_λ550_ was found to decrease from 64.4% to 46.7% as the doping concentration increased from 1.3 at.% to 8.1 at.%. As the doping concentration increased, the films exhibited a stronger blue coloration, a characteristic widely noted in Sb‐doped SnO_2_ films [60, 61, 62]. Several explanations have been proposed to account for this observation in relation to the opto‐electronic properties of the films. One explanation is that increasing the Sb doping concentration results in a higher number of excitable electrons, which is attributed to the greater presence of Sb^5+^ [62].

Upon Sb‐doping, the most pronounced changes occurred in the near‐infrared (NIR,750‐1400 nm) and short‐wave infrared (SWIR, 1400–2500 nm) regions and were attributed to free‐carrier absorption (Drude effect) [2, 63]. Undoped SnO_2_ showed T_λ750‐1400_ of 82.3%, but Sb doping reduced it to 35%–38% in **ATO‐1** to **ATO‐3** and to only 15% in **ATO‐4**. A similar trend was observed in the SWIR region, where undoped SnO_2_ transmitted 72.0% in T_λ1400‐2500_, while **ATO‐4** transmitted only 0.14%, effectively blocking most of the infrared. Such strong infrared absorption is a characteristic of functional TCO materials and is desirable in solar‐control and heat‐shielding coatings.

The film deposited using the same solution chemistry as **ATO‐2** but with a longer misting time (ATO‐5, 10 min) exhibited a reduced transmittance at 550 nm (T_λ550_ = 56.5%). This decrease can be attributed to the combined effects of increased film thickness, enhanced light scattering, and stronger free‐carrier absorption. In the NIR and SWIR regions, **ATO‐5** transmitted more light (44.3% and 15.4%, respectively) than the heavily doped **ATO‐4**, reinforcing that both dopant concentration and film thickness critically determine the spectral selectivity of ATO films. **ATO‐1**‐**ATO‐4** showed SWIR transmittance of <3% and hence they can find applications in heat‐mirror coatings or heat‐shielding glazing where SWIR blocking is desired [64].

The pronounced ultraviolet absorption observed in all films highlights their potential for use as functional coatings in smart windows and display panels, where they can provide protection to sensitive materials and human eyes against harmful UV radiation [65].

The Percent Haze Was Calculated by Using Equation 3 [66]

(3) $$\mathrm{Haze}\;(\%)\;=\frac{\mathrm{Diffuse}\;\mathrm{transmittance}}{\mathrm{Total}\;\mathrm{transmittance}}\;\times 100$$

**FIGURE 7:**
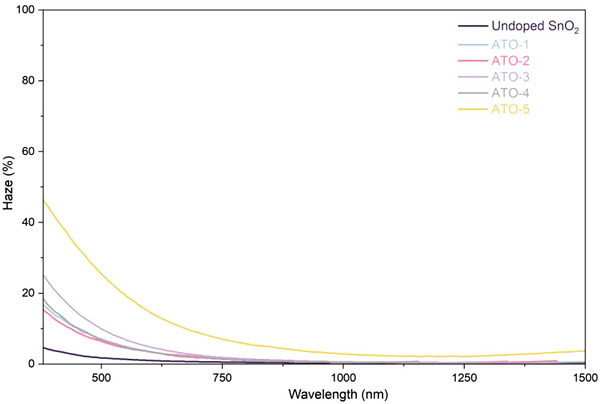
Haze spectra of undoped SnO_2_ and ATO samples.

The haze spectra of all films are shown in Figure 7, and the average haze values in the visible region (380–750 nm) are listed in Table 2. The haze values were found to increase upon Sb doping, with a noticeable rise in haze as the Sb concentration in the films increased. Similar increases in optical haze or diffuse scattering with increasing Sb incorporation have been reported for ATO thin films and are attributed to enhanced light scattering arising from dopant‐induced microstructural distortion [66, 67]. **ATO‐2** exhibited a haze value of 5.5%, whereas its thicker analogue, **ATO‐5**, showed a significantly higher haze value of 21%. An increase in haze with thickness has been previously reported [66]. Overall, **ATO‐1** to **ATO‐4** films exhibited haze values below 10%, indicating that the films preserved optical clarity alongside infrared shielding, which is a key requirement for low‐emissivity architectural glazing where minimal visual distortion and effective solar control are essential [67].

**FIGURE 8:**
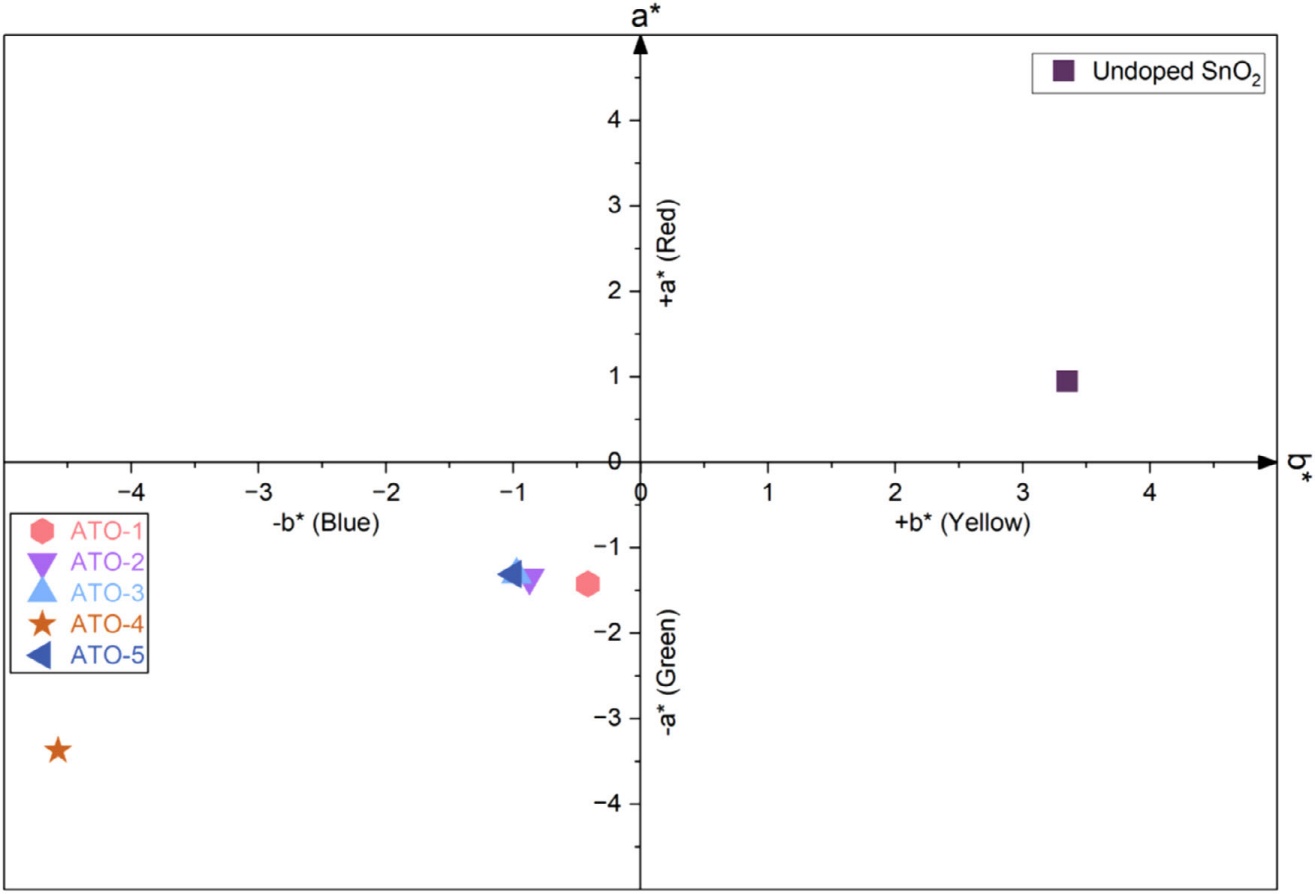
CIE chromaticity diagram showing the color coordinates (a* and b*) of undoped SnO_2_ and ATO films.

The color evolution of the films with Sb‐concentration and the corresponding chromaticity coordinates are shown in Figure 8. The details of the calculations are provided in the Supporting Information. The undoped film exhibited a nearly neutral appearance, whereas Sb incorporation resulted in a systematic shift toward negative b* values, indicating the development of a bluish tint. The color positions and corresponding lightness values for each sample are provided in Figure S4, which further illustrate the gradual bluish darkening of the films with Sb‐concentration.

**ATO‐2** and **ATO‐5** showed similar a* and b* values despite the greater thickness of **ATO‐5**, indicating that the blue coloration was primarily governed by Sb concentration rather than film thickness. In contrast, the lower L* value of **ATO‐5** (Figure S4) showed that increased thickness mainly influenced the film lightness, resulting in a darker appearance without significantly altering chromaticity. Overall, these results suggested that the blue coloration of the films can be effectively tuned by changing the Sb concentration.

**FIGURE 9:**
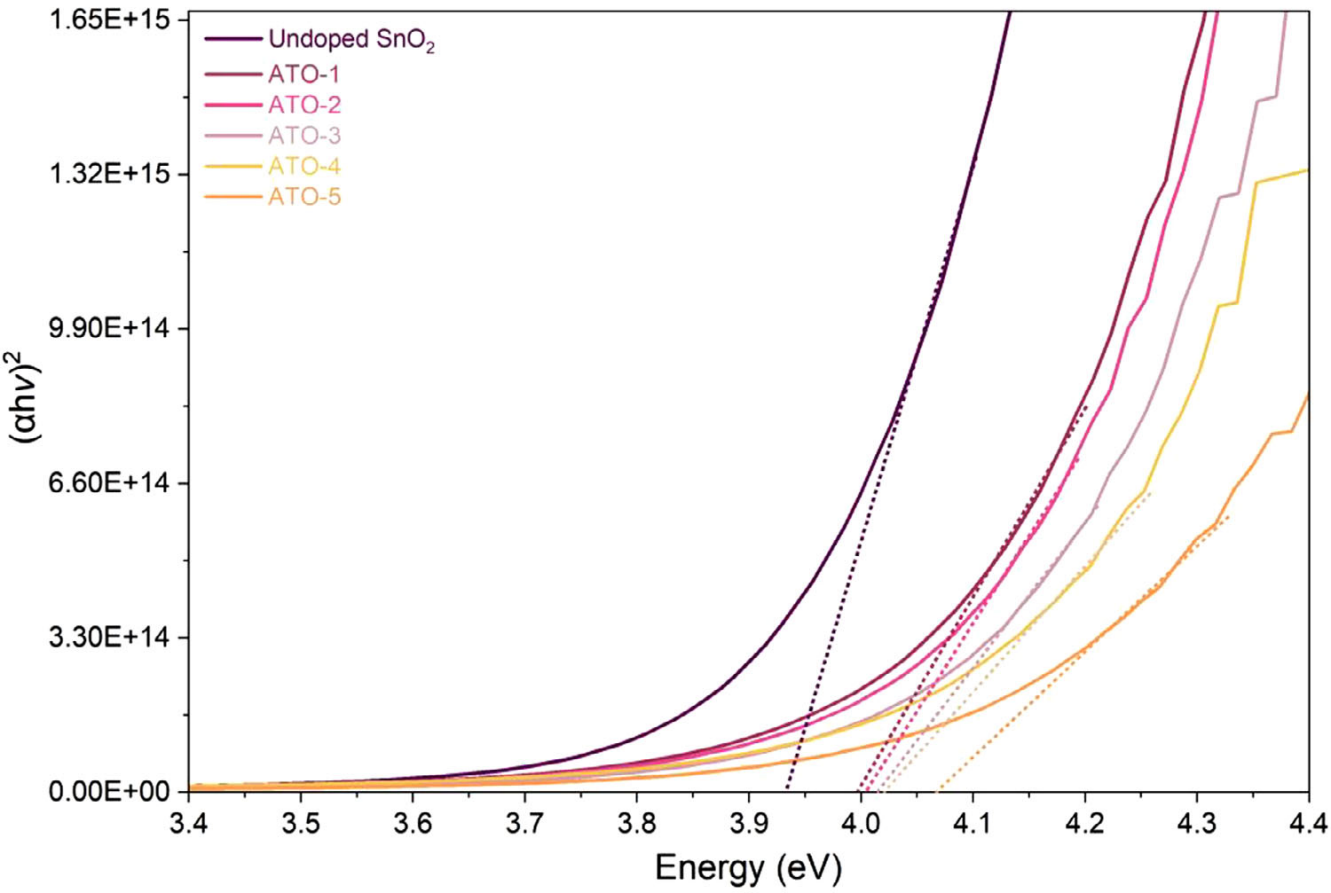
Tauc plots of undoped SnO_2_ and ATO samples.

The optical bandgap (E_g_) values were calculated using the Tauc method [68], and shown in Figure 9. In this method, the linear region of a plot of (*αhν*)^2^ versus Energy E_g_ (eV) near the high‐energy absorption edge was extrapolated to the x‐axis to provide an estimate of the bandgap energy. In the given Tauc method (Equation 4) [68], α represents the linear absorption coefficient, *A* is a constant, and *hν* denotes the photon energy, where *h* is Planck's constant and ν is the frequency of the incident light.

(4) $${\left(\alpha h\nu \right)}^{2}=A\left(h\nu -E_{g}\right)$$

The optical bandgap of the undoped SnO_2_ film was determined to be 3.93 eV, which is consistent with reported values for polycrystalline SnO_2_ [53, 54]. Upon Sb‐doping, a widening of the bandgap was observed with values ranging from 3.99 to 4.06 eV across the doped films. This suggests the influence of the donor levels of dopant in inducing degenerate semi‐conductivity and widening the optical bandgap, as explained by the Moss‐Burstein effect, where the filling of lower conduction band levels leads to an increase in the bandgap [3, 69].

For instance, **ATO‐1** and **ATO‐2** exhibited bandgaps of 3.99 and 4.0 eV, respectively, consistent with their increased carrier concentrations (3.8 × 10^20^ and 1.0 × 10^21^ cm^−3^). The effect was most evident in **ATO‐5**, which showed the widest bandgap (4.06 eV). Despite having only moderate transmittance at 550 nm (56.5%), this film showed high crystallinity and the highest charge carrier concentration (3.6 × 10^21^ cm^−3^).

Hall Effect measurements at room temperature were performed in the Van der Pauw configuration on the most conductive areas of all tested films to evaluate their electrical conductivity. The results are presented in Table 2. The negative Hall coefficient values confirmed the n‐type conductivity of the films. It is well known that the physical properties of oxides are strongly influenced by deviations from stoichiometric composition (native disorder) and by the nature and concentration of foreign atoms incorporated into the crystal lattice.

The films were stored in air under ambient conditions for 15 months, during which no noticeable degradation or measurable changes in electrical properties were observed. The films (**ATO‐1** and **ATO‐2**) were subjected to 20 cycles of Scotch tape testing, and no visible edge peeling, delamination, or surface damage was detected. Electrical measurements carried out after the tape tests showed no measurable change in resistivity, indicating that the electrical performance of the films was maintained. In addition, the films showed good resistance to mechanical damage during manual scratching tests performed using a stainless‐steel spatula.

Since tin oxide is an n‐type semiconductor, oxygen vacancies (V_o_) or interstitial tin atoms (Sn_i_) are expected to act as donors in pure SnO_2_ [70, 71]. The SnO_2_ films appeared to be oxygen‐deficient (Table 1). According to the literature, the formation of oxygen vacancies on the surface and/or subsurface regions of the growing SnO_2_ occurs under the applied deposition conditions, specifically at a high temperature of above 500°C [72].

The undoped SnO_2_ film exhibited a resistivity of 8.9 × 10^−4^ Ω cm, mobility of 21.5 cm^2^ V^−1^ s^−1^, and carrier concentration of 3.3 × 10^20^ cm^−3^. This low resistivity for undoped SnO_2_ is comparable to that of doped SnO_2_ films, as shown in Table 3 [8, 9, 73, 74]. However, similarly low resistivity values of the same order of magnitude have also been reported in the literature for Undoped SnO_2_ films deposited by other deposition methods [10, 11, 12, 13].

**TABLE 3:** Optoelectronic properties of Sb‐doped SnO_2_ films previously reported using Chemical Vapor Deposition (CVD) methods.

Deposition Method	Resistivity (Ω cm)	Mobility (cm^2^ V^−1^ s^−1^)	Carrier Conc. (cm^−3^)	Transmittance (%)	Thick ness (nm)	Bandgap (eV)	Sheet Resistance (Ω □^−1^)	References
MOCVD^a^	1.3 × 10^−3^	9.0	2.5 × 10^20^	80	150	4.11	—	[16]
AACVD	4.7 × 10^−4^	11.4	1.2 × 10^21^	65	525	—	9.0	[53]
AACVD	1.0 × 10^−3^	—	—	70	30	—	105	[52]
MOCVD	7.63 × 10^−4^	19.9	4.1 × 10^20^	80	—	—	—	[54]
Mist CVD	9.72 × 10^−4^	8.0	1 × 10^21^	95	220	4.41	44.1	[15]
Mist CVD	6.58 × 10^−4^	2.9	3.2 × 10^21^	90	190	4.22	34.6	[51]
APCVD^a^	3.38 × 10^−2^	23.5	7.7 × 10^17^	44	320	3.74	1056	[14]

^a^ MOCVD—Metal‐organic CVD, APCVD—Atmospheric Pressure CVD.

Upon Sb‐doping, the electrical properties were strongly modified reflecting an interplay between enhanced donor density and increased carrier scattering [21]. Resistivity decreased at moderate Sb levels, reaching 5.3 × 10^−4^ Ω cm in **ATO‐2**. This trend is driven by a substantial increase in carrier concentration from 3.2 × 10^20^ cm^−3^ (undoped) to 1.0 × 10^21^ cm^−3^ (**ATO‐2**), while mobility declined from 23.0 to 11.1 cm^2^ V^−1^ s^−1^. A further increase in carrier concentration was achieved for **ATO‐3** (1.3 × 10^21^ cm^−3^). However, a pronounced reduction in mobility to 3.7 cm^2^ V^−1^ s^−1^ led to an increased resistivity of 1.3 × 10^−3^ Ω cm, indicating the onset of scattering.

To assess the contribution of grain‐boundary scattering, the carrier mean free path was estimated from the Hall carrier concentration and resistivity data (see Supporting Information for detailed calculations) [75]. The calculated carrier mean free paths ranged from 0.34 to 3.03 nm, substantially smaller than the corresponding crystallite sizes of 28.1–36.9 nm (Table 1). This indicates that charge carriers undergo multiple scattering events within individual crystallites before reaching a grain boundary. Therefore, grain‐boundary scattering is unlikely to be the dominant mobility‐limiting mechanism in these films. Instead, the reduction in mobility with increasing Sb concentration is consistent with enhanced scattering arising from ionized impurities and structural disorder associated with heavy doping [75].

**FIGURE 10:**
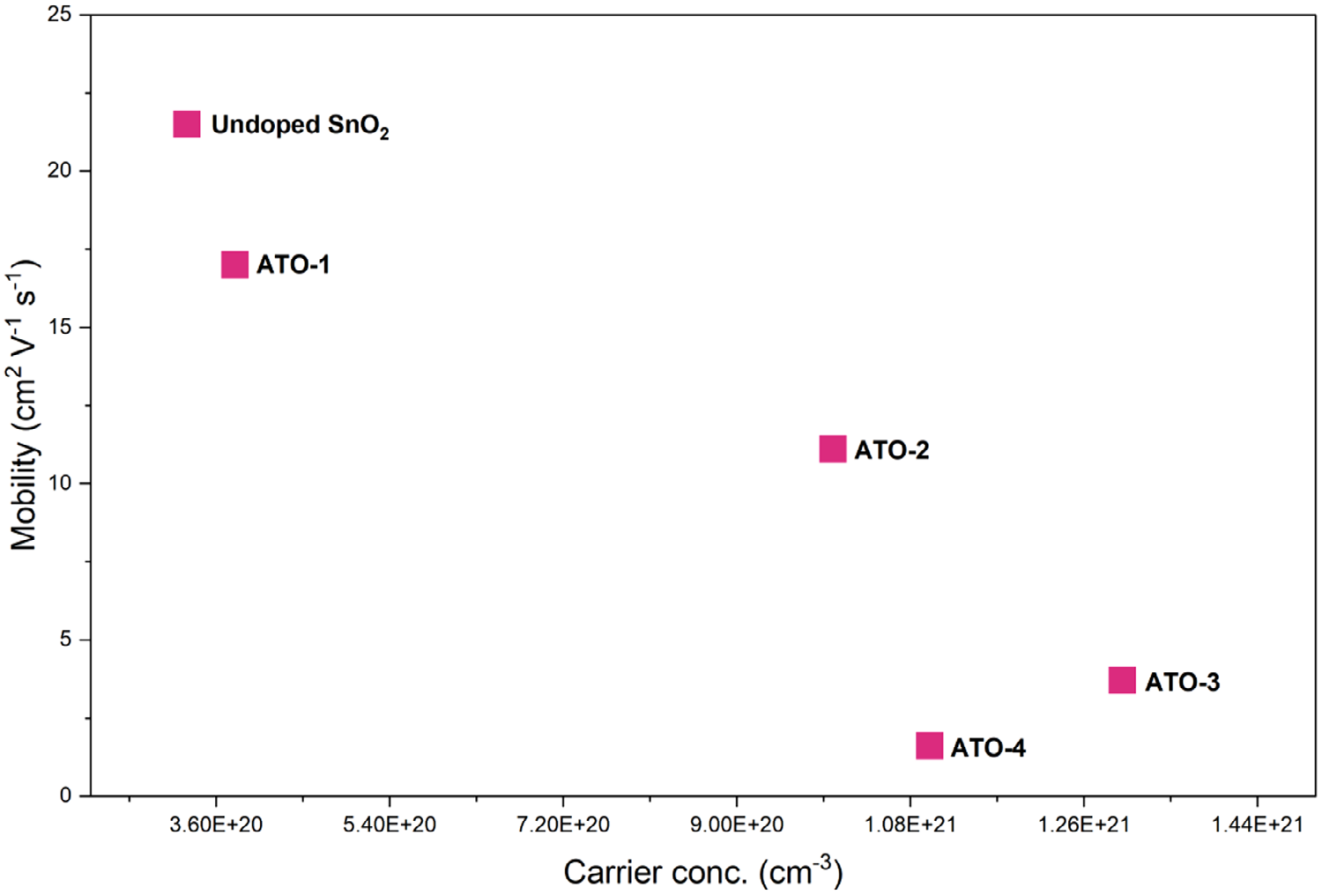
Mobility vs. carrier concentration for undoped SnO_2_ and ATO films.

At higher Sb concentrations, carrier scattering effects became dominant. **ATO‐4**, containing the highest Sb content, exhibited a severely reduced mobility of 1.6 cm^2^ V^−1^ s^−1^ and a correspondingly high resistivity of 3.9 × 10^−3^ Ω cm, despite maintaining a high carrier concentration of 1.1 × 10^21^ cm^−3^. This behavior highlighted the detrimental impact of excessive dopant incorporation, where defect‐induced scattering and microstructural disorder outweighed the benefits of increased charge carriers. This behavior is further shown in Figure 10, which shows the relationship between carrier concentration and mobility for the undoped and ATO films. A clear inverse trend is observed, where mobility decreases with increasing carrier concentration. As Sb^5+^ incorporation introduces additional free electrons, it simultaneously increases the density of charged scattering centers, limiting carrier mobility. At higher doping levels, this effect becomes dominant, indicating that further increases in carrier concentration do not translate into improved electrical performance due to enhanced scattering and possible microstructural disorder.

Similar trends of resistivity decreasing at moderate Sb doping and increasing at higher concentrations have been widely reported [14, 15, 16, 21]. These resistivity values are also lower than those of Sb:SnO_2_ films reported via spray pyrolysis using the same tin precursor (SnCl_4_) [9, 73]. The electronic properties of Sb:SnO_2_ films previously reported using CVD methods, as shown in Table 3 [14, 15, 16, 51, 54].

Sheet resistance reflects the balance between resistivity and film thickness. The undoped film (463 nm) showed a sheet resistance of 18.4 Ω □^−^^1^, while **ATO‐2** (686 nm) showed a significantly reduced value of 7.7 Ω □^−^^1^, reflecting its improved electrical conductivity. These values are lower than those reported in the literature [9, 21, 73]. A noticeable thickness effect was evident for **ATO‐5**, which was deposited using the same solution chemistry as **ATO‐2** but with extended misting time. Despite similar Sb doping levels, the increased thickness of 920 nm resulted in a very low sheet resistance of 2.2 Ω □^−^^1^, combined with a high carrier concentration of 3.6 × 10^21^ cm^−3^ and a low resistivity of 2.0 × 10^−4^ Ω cm. Similar results for F‐doped tin oxide films are reported [35].

Residual chlorine and oxygen stoichiometry also played important roles. XPS analysis revealed chlorine contents between 0.6 and 0.8 at.%. Chlorine in SnO_2_ can occupy two possible states: (i) substitutional Cl^−^ at oxygen sites, where it can act as a shallow donor analogous to fluorine [36], and (ii) grain‐boundary or adsorbed chloride species, which introduce charged centers that scatter carriers [76].

In the present series, the chlorine levels are relatively low and with narrow range but still show correlations with mobility trends. For example, **ATO‐2** contained ∼0.8 at.% Cl, coinciding with a reduction in mobility to 11.1 cm^2^ V^−1^ s^−1^, despite its favorable carrier density (1.0 × 10^21^ cm^−3^). In contrast, **ATO‐5**, with ∼0.6 at.% Cl, achieved the highest electron concentration (3.6 × 10^21^ cm^−3^) and one of the lowest sheet resistances, consistent with reduced scattering at lower chlorine levels. Therefore, chlorine was regarded as a secondary but non‐negligible contributor to the electrical response.

In a previous study on optimization of undoped SnO_2_ films, the level of chlorine incorporation was systematically investigated and controlled by varying deposition parameters such as solvent composition and deposition temperature [77]. In the present study, all depositions were carried out at a relatively high temperature (590°C) using optimized solvent ratios. Under these conditions, chlorine was consistently detected at levels below 1 at.% in all films and was not observed to vary with Sb doping. Therefore, the chlorine concentration in both undoped and doped films remained effectively constant, and the observed changes in optoelectronic properties upon doping are primarily attributed to Sb incorporation. These results indicated that antimony was incorporated into SnO_2_ films that already contained residual chlorine originating from the chloride precursor. Notably, the chlorine concentration did not increase significantly upon the introduction of SbCl_5_ as the dopant precursor, suggesting that the residual chlorine present in the films predominantly originated from the SnCl_4_ precursor. Similar observations are previously reported by Messad et al. for Sb‐doped SnO_2_ films deposited using SnCl_4_ and SbCl_3_ precursors, where residual chlorine incorporation was attributed to the SnCl_4_ source [78]. The presence of residual chlorine in SnO_2_ films deposited using SnCl_4_‐based precursors has been widely reported in the literature [79, 80].

Similarly, the O/Sn ratio provides insight into the role of oxygen vacancies. All the films were found to be oxygen deficient, as shown in Table 1. The low resistivity of SnO_2_ films due to oxygen vacancies has been widely reported in the literature [71, 81, 82, 83]. Mukhamedshina et al. reported a decrease in surface resistance when films were treated with hydrogen plasma, attributing this effect to the presence of oxygen vacancies [81]. The lowest O/Sn ratio (1.5 in **ATO‐4**) corresponded to high carrier density but also the strongest mobility degradation. However, the relatively oxygen‐rich **ATO‐5** (O/Sn = 1.9) still achieved the highest carrier concentration, which reinforced that Sb^5+^ substitution as the dominant donor mechanism.

Superior transport properties such as high carrier concentration are often achieved at the cost of reduced optical transmittance. To assess the optoelectronic performance of TCO materials the figure of merit (F.O.M.) proposed by Haacke in 1976 [84] was employed in this study. The F.O.M. (Φ) is defined in Equation 5.

(5) $$\Phi =\frac{T^{10}}{R_{sh}}\;$$

**ATO‐2** showed a favorable balance between conductivity and visible transmittance, yielding a F.O.M. of 2.4 × 10^−3^ Ω^−1^. **ATO‐4** showed a drastically reduced F.O.M. of 0.01 × 10^−3^ Ω^−1^, primarily due to its poor optical transmittance and high sheet resistance. However, **ATO‐5** achieved an enhanced F.O.M. of 1.5 × 10^−3^ Ω^−1^, demonstrating that thickness‐driven conductivity enhancement can partially recover optoelectronic performance. Overall, Sb^5+^ doping is the principal driver of conductivity improvement in these films, while chlorine and oxygen stoichiometry modulate scattering and compensation effects. The comparison between **ATO‐2** and its thicker analogue **ATO‐5** showed that thickness can be used as an effective parameter to further reduce sheet resistance. The reproducibility of the films was verified by preparing samples (**Undoped SnO_2_**, **ATO‐1** and **ATO‐4**) three times, which consistently yielded nearly consistent electrical properties and film thickness.

**FIGURE 11:**
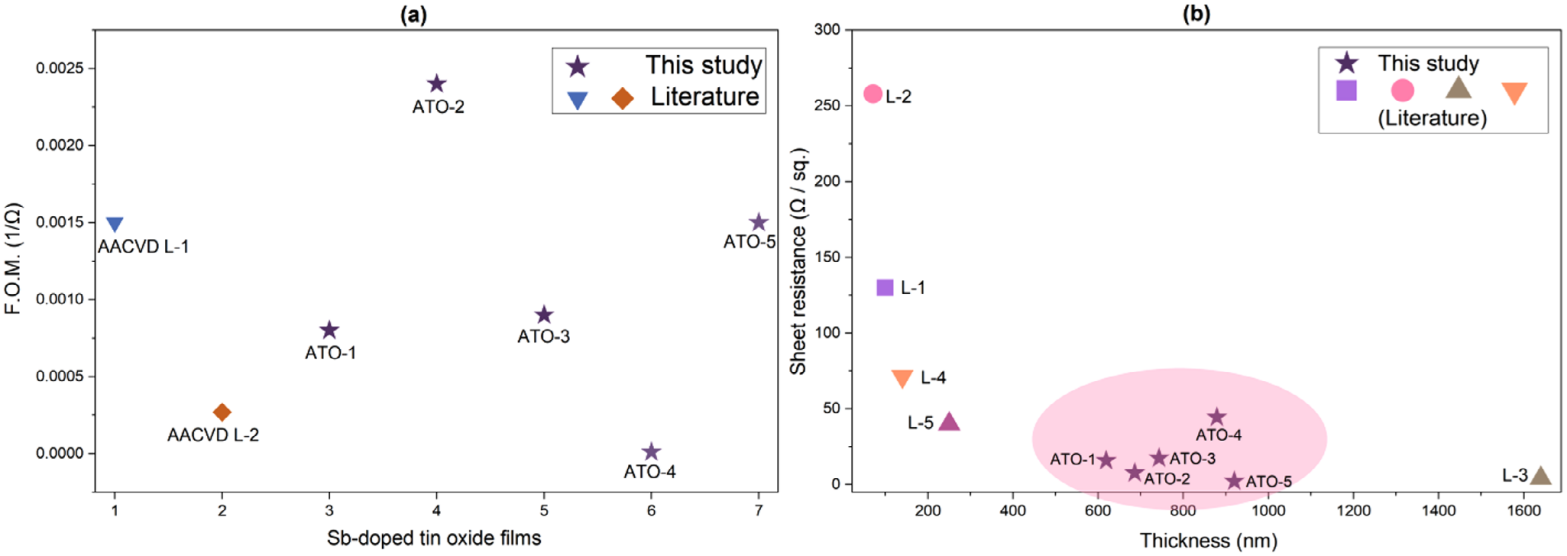
Comparison of the performance of ATO films prepared in this work with previously reported literature: **(a)** Comparison of the F.O.M of films from this study with ATO films deposited via AACVD reported in the literature **(b)** Comparison of sheet resistance as a function of film thickness for films from this study and literature ATO films deposited using SnCl_4_ as a precursor. (references: AACVD L‐1 [21], AACVD L‐2 [52] and L‐1 [85], L‐2 [86], L‐3 [87], L‐4 [88], and L‐5 [89]).

The electronic properties of the films presented in this work are comparable to those of Sb‐doped SnO_2_ films previously reported using CVD methods, as shown in Table 3 [14, 15, 16, 53, 54]. This comparison was further extended by comparing the F.O.M. of the films deposited in this study with previously reported ATO films deposited by AACVD (Figure 11a), and by comparing the sheet resistance and film thickness of films from this study with ATO films reported in the literature that were deposited using SnCl_4_ as a precursor (Figure 11b). The F.O.M. comparison was not possible in Figure 11b, as most literature reports did not explicitly state transmittance values.

As shown in Figure 11a, the F.O.M. for **ATO‐2** (this study) was higher than that reported for ATO films deposited via AACVD. This enhanced performance can be attributed to the combination of low sheet resistance and moderate optical transmittance, indicating efficient carrier transport without significant optical loss. The remaining films (**ATO‐1**, **ATO‐3**, **ATO‐4** and **ATO‐5**) showed F.O.M. values comparable to those reported in the AACVD literature.

Literature reports on ATO films deposited using SnCl_4_ as a precursor (Figure 11b) showed a wide distribution of sheet resistance values over a broad thickness range. The films prepared in this work occupied a region of comparable or lower sheet resistance, indicating efficient electrical transport without the need for significantly thicker films. Notably, **ATO‐2** and **ATO‐5** achieved the lowest sheet resistance values while maintaining moderate thickness, suggesting improved film continuity and carrier transport properties. Overall, the results suggest that precursor chemistry, deposition conditions and tuning of dopant concentration enabled the fabrication of ATO films with optimized optoelectronic performance.

Overall, the results demonstrate that the deposition conditions employed in this study enable the fabrication of ATO films with competitive optoelectronic performance relative to previously reported AACVD and SnCl_4_‐derived ATO films. The observed improvements highlight the effectiveness of the present growth approach in achieving a favorable trade‐off between conductivity and transparency, which is essential for transparent conducting oxide applications.

**FIGURE 12:**
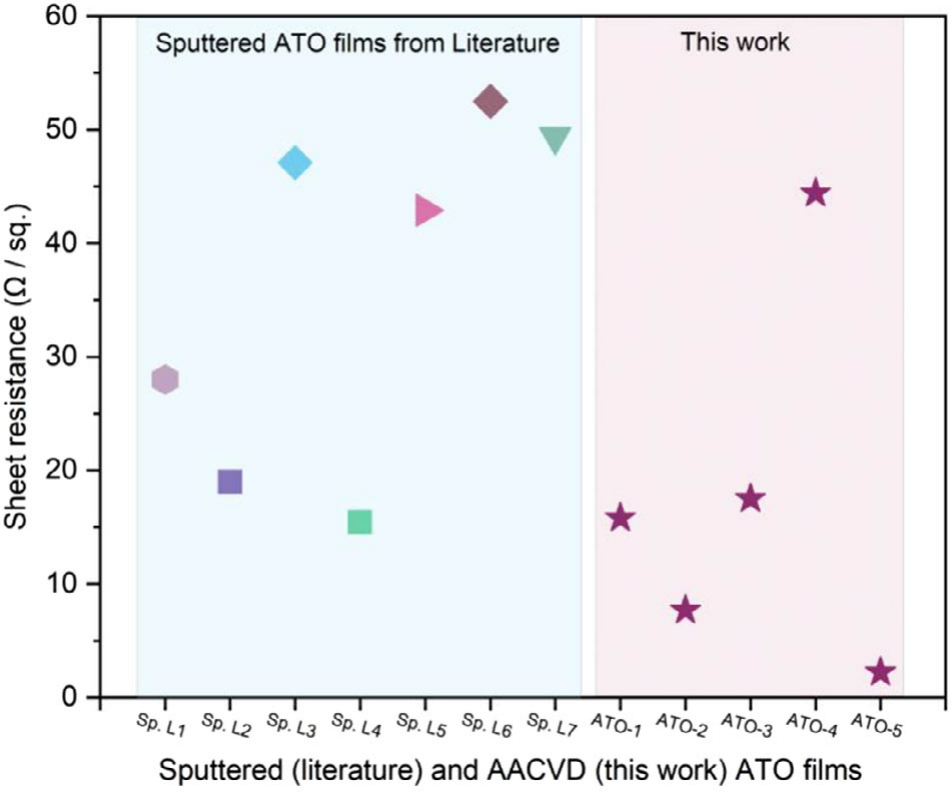
Comparison of sheet resistance for sputtered ATO films reported in the literature (Sp. L1 [90] Sp. L2 [91], Sp. L3 [92], Sp. L4 [93], Sp. L5 [94], Sp. L6 [95], and Sp. L7 [96]) and AACVD‐deposited ATO films from this work.

The sheet resistance of the deposited films is also compared with reported ATO films deposited via the widely used sputtering technique, as shown in Figure 12. The AACVD‐deposited films prepared in this work are shown to exhibit sheet resistance values that are comparable to those reported for sputtered ATO films. Lower sheet resistance values are achieved for **ATO‐2** and **ATO‐5** compared to the sputtered films included in this study. These results indicate that ATO films with electrical performance comparable to, and in some cases superior to, sputtered films can be produced via AACVD.

**FIGURE 13:**
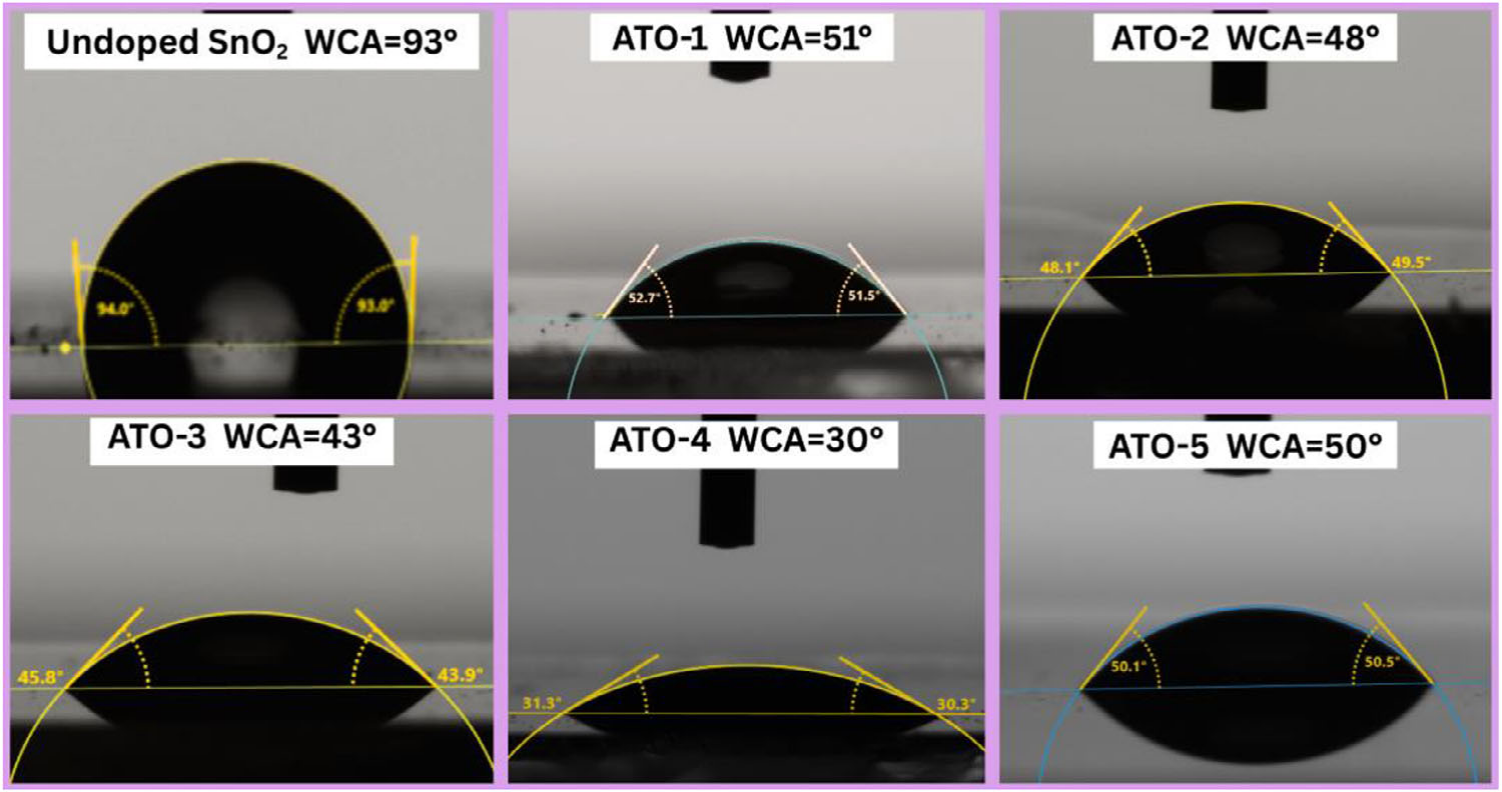
Water contact angle (WCA) images of undoped and Sb‐doped SnO_2_ samples.

The static water contact angle (WCA) measurements showed a pronounced wettability shift upon Sb‐doping, as shown in Figure 13. The undoped SnO_2_ showed borderline hydrophobicity (93°), whereas all Sb‐doped films were hydrophilic. A similar decrease in the WCA of SnO_2_ films upon Sb doping has been reported previously [97]. From **ATO‐1** to **ATO‐4** the WCA decreased progressively from 51° to 30°. This behavior is consistent with Sb‐driven modification of the surface defect chemistry i.e. XPS O 1s deconvolution identified a higher binding energy contribution assigned to surface hydroxyl groups (─OH). Hydroxylated or polar surfaces has already been reported to increase the wettability [98]. Within this sample set, the strongest hydrophilicity was observed for ATO‐4 (WCA 30°), which is also the most heavily doped film by XPS (Sb = 8.1 at.%). It also exhibited the lowest O/Sn ratio (1.5), i.e., the greatest oxygen deficiency among the series. Oxygen‐deficient oxide surfaces promote dissociative adsorption of water and stabilization of hydroxyl species, which increases the polar component of the surface energy and lowers WCA [99].

**ATO‐2** and **ATO‐5** exhibited nearly identical WCAs (48° and 50°, respectively), suggesting that increasing misting time did not significantly modify the surface wettability. This further suggested that the wetting behavior was governed primarily by the surface chemistry rather than by bulk thickness. Previously, it has been reported that variations in film thickness do not significantly affect the WCA [100].

## 4 Conclusions

Antimony‐doped SnO_2_ thin films were deposited by AACVD and systematically characterized for their structural, electrical, and optical performance. X‐ray diffraction confirmed crystallization in the cassiterite SnO_2_ phase without secondary phases, demonstrating successful incorporation of Sb into the lattice. Moderate Sb doping significantly enhanced electrical conductivity, with **ATO‐2** showed a resistivity of 5.3 × 10^−4^ Ω cm, a carrier concentration of 1.0 × 10^21^ cm^−3^, and a mobility of 11.1 cm^2^ V^−1^ s^−1^, reflecting effective activation of Sb^5+^ donors. At higher Sb concentrations, increased ionized impurity and grain‐boundary scattering reduced mobility and electrical performance despite high carrier densities. XPS revealed residual chlorine (<1 at.%), which contributed both shallow donor states and charged scattering centers, while oxygen vacancies acted as additional intrinsic donors. Film thickness was identified as an important parameter for electrical optimization, as **ATO‐5** achieved a low resistivity of 2.0 × 10^−4^ Ω cm and a sheet resistance of 2.2 Ω □^−1^ through extended deposition time without excessive Sb incorporation. Optical measurements showed acceptable visible transparency, low haze values (<10% for **ATO‐1** to **ATO‐4**) indicating preserved optical clarity accompanied by pronounced infrared blocking due to free‐carrier absorption and tuneable blue‐grey tint. CIE *L*a*b* colorimetric analysis further confirmed a gradual shift toward negative *b* values with increasing Sb concentration, demonstrating tuneable blue colouration governed primarily by dopant incorporation. These combined properties demonstrate that AACVD deposited ATO films are promising multifunctional coatings for low‐emissivity and solar‐control architectural glazing, infrared heat‐shielding windows, transparent electrodes and other energy‐efficient optoelectronic applications.

## Author Contributions

**Iqra Ramzan** prepared and characterized the samples and contributed to the writing and editing of the manuscript. Claire Carmalt and Ivan Parkin supervised the project and contributed to writing and editing. All authors participated in scientific discussions throughout the study.

## Conflicts of Interest

The authors declare no conflicts of interest.

## Supporting information




**Supporting File**: advs76476‐sup‐0001‐SuppMat.docx.

## Data Availability

The data that support the findings of this study are available from the corresponding author upon reasonable request.
