# Supplementary material for: Optimized Antimony‐Doped Tin Oxide Thin Films With Enhanced Electrical Properties for Low‐Emissivity Architectural Coatings
Source: Adv Sci (Weinh). 2026 Jul 9:e76476. Online ahead of print. doi: 10.1002/advs.76476 (PMC13348347; doi:10.1002/advs.76476)
Supplement: Supplementary file 1 — Supporting File: advs76476‐sup‐0001‐SuppMat.docx. [file ADVS-9999-e76476-s001.docx]

**Supporting Information**

**Optimised Antimony-Doped Tin Oxide Thin Films with Enhanced Electrical Properties for Low-emissivity Architectural Coatings**

*Iqra Ramzan^a^, Ivan P. Parkin^a^, and Claire J. Carmalt^a^**

^a^Materials Chemistry Centre, Department of Chemistry, University College London, 20 Gordon Street, London WC1H 0AJ, UK.

***Corresponding Author’s Email: c.j.carmalt@ucl.ac.uk


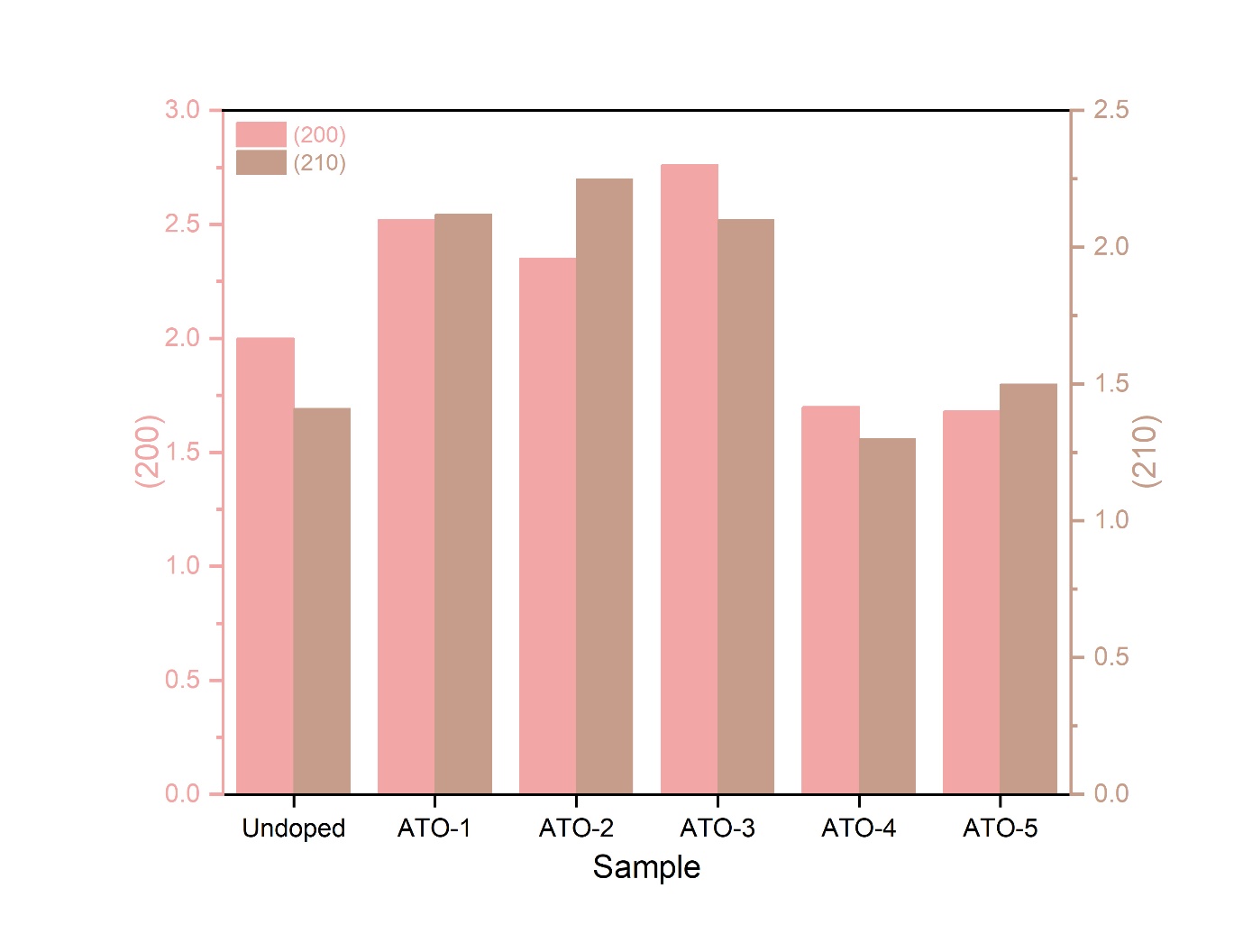


**Figure S1.** Texture coefficient (TC) values of all the films for preferred (200) and (210) planes


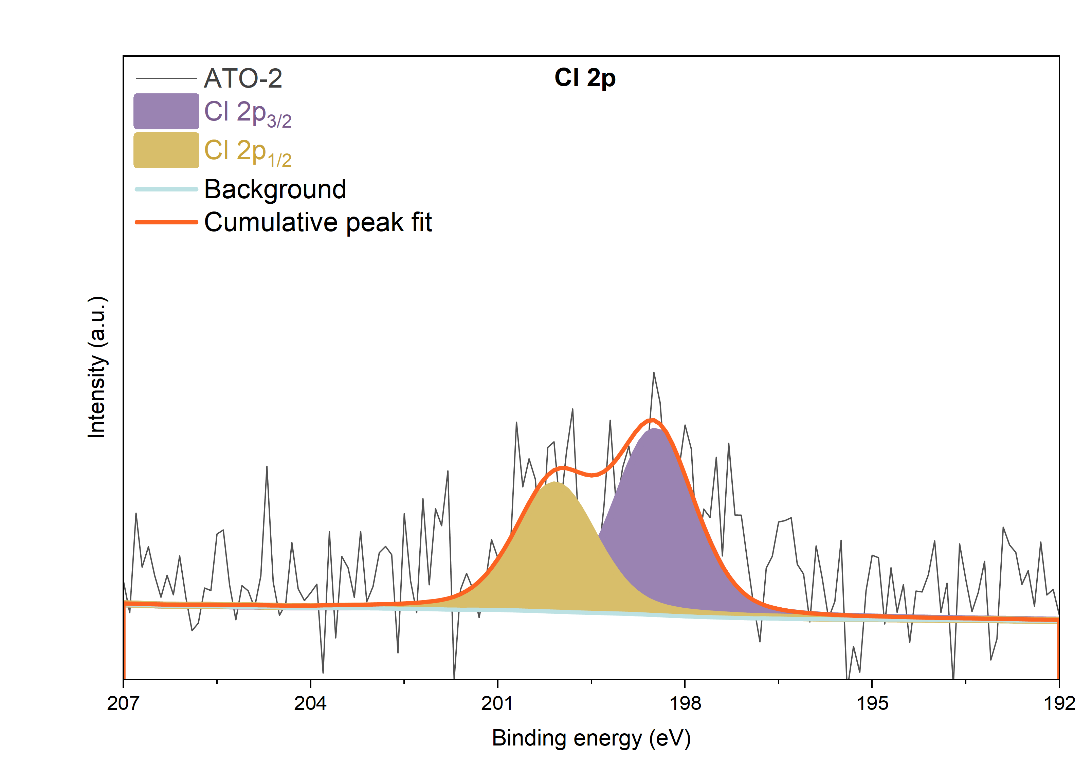


**Figure S2.** Deconvoluted Cl 2p spectrum of sample **ATO-2**


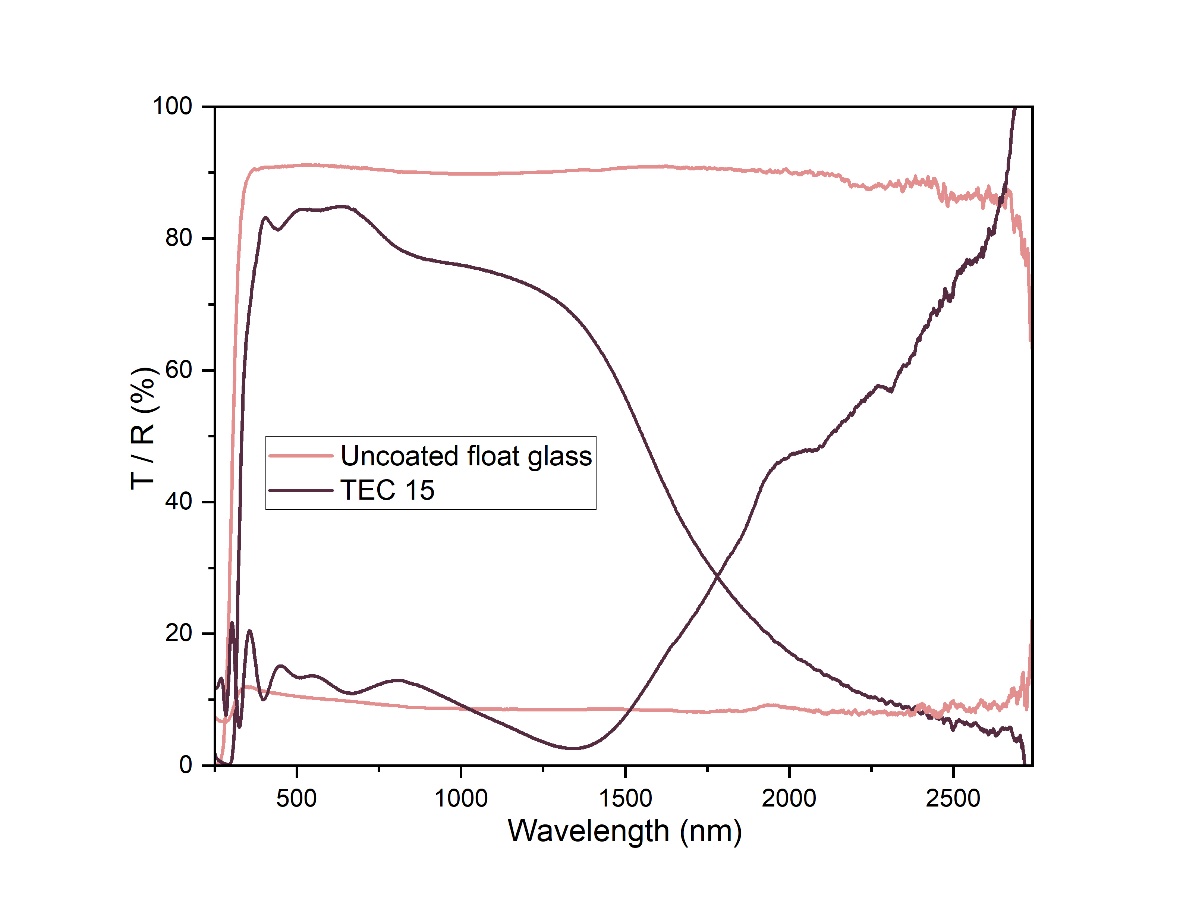


**Figure S3.** Transmittance and reflectance spectra of uncoated float glass and TEC-15 (from NSG Pilkington)

The colour appearance of the films was quantified using CIE colourimetric analysis based on optical transmittance data. Spectral transmittance measured over the wavelength range 360-780 nm was used to calculate colour coordinates under the CIE standard illuminant D65 (representing daylight conditions) together with the CIE 10° standard observer function.^[1,2]^

The tristimulus values (X, Y, Z) were calculated by integrating the wavelength-dependent transmittance with the spectral power distribution of the D65 illuminant and the CIE 10° colour-matching functions according to standard colourimetric relations (**Equations 1**-**3**).^[3]^

$X=k\sum T(\lambda)S(\lambda)x(\lambda)$  **(1)**

$Y=k\sum T(\lambda)S(\lambda)y(\lambda)$ **(2)**

$Z=k\sum T(\lambda)S(\lambda)z(\lambda)$ **(3)**

Where $T(\lambda)$is the measured transmittance, $S(\lambda)$is the spectral power distribution of the D65 illuminant, and $x(\lambda)$, $y(\lambda)$, and $z(\lambda)$ are the CIE colour matching functions. The normalisation constant $k$was applied such that a perfect transmitting diffuser under the same illumination yields $Y=100$.

The calculated tristimulus values were subsequently normalised using the reference white coordinates $X_{n},Y_{n},Z_{n}$ corresponding to the D65 illuminant. The CIE Lab* colour coordinates were then obtained using the standard nonlinear transformation (**Equations 4**-**6**).

$L^{*}=116f(Y/Y_{n})-16$ **(4)**

$a^{*}=500[f(X/X_{n})-f(Y/Y_{n})]$ **(5)**

$b^{*}=200[f(Y/Y_{n})-f(Z/Z_{n})]$  **(6)**

Where $f(t)$ represents the CIE nonlinear response function describing human visual perception.

The parameter $L^{*}$ represents lightness, while $a^{*}$ and $b^{*}$ describe chromaticity along the red-green and yellow-blue axes, respectively. Negative $b^{*}$ values indicate blue coloration, whereas positive $b^{*}$ values correspond to yellow hues.


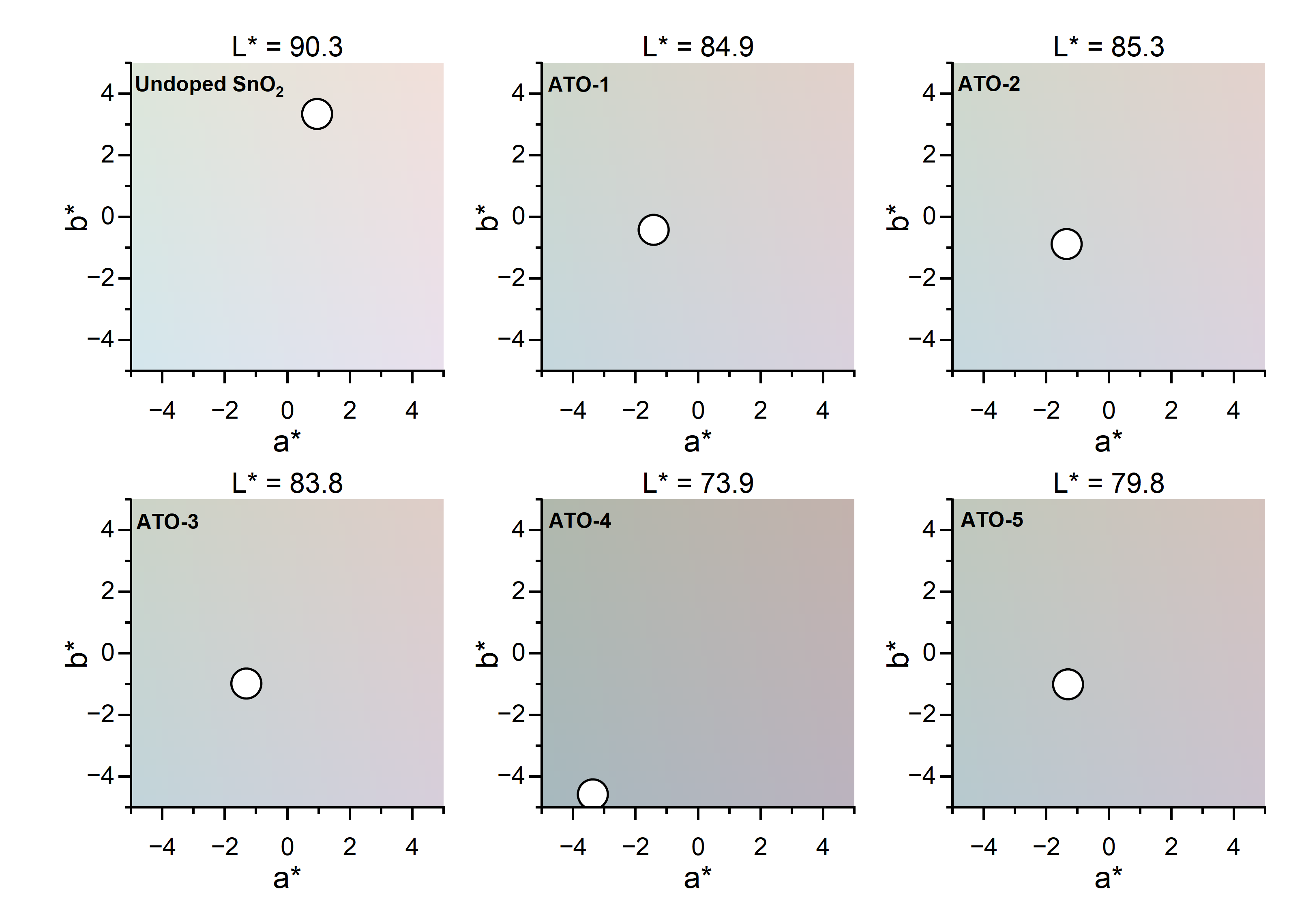


**Figure S4.** CIE *Lab** colour coordinates of undoped and ATO films showing the corresponding lightness (*L*⁎) and chromaticity positions in the *a**-*b** plane

**Carrier Mean Free Path Calculation**

The carrier mean free path ($l_{mfp}$) was estimated following the approach reported by Jia *et al*. for doped SnO_2_ thin films.^[4]^ The mean free path was calculated using:

$l_{mfp}=V_{F}\tau$ **(7)**

where $V_{F}$ is the electron velocity at the Fermi surface and $\tau$ is the carrier relaxation time. The Fermi velocity is given by:

$V_{F}=\frac{\hbar(3\pi^{2}n)^{1/3}}{m^{*}}$ **(8)**

where $\hbar$ is the reduced Planck constant, $n$ is the Hall carrier concentration and $m^{*}$ is the electron effective mass. The relaxation time was estimated from the Drude model according to:

$\tau=\frac{m^{*}}{ne^{2}\rho}$ **(9)**

where $e$ is the elementary charge and $\rho$ is the electrical resistivity. Substituting the expressions for $V_{F}$ and $\tau$ yields:

$l_{mfp}=\frac{\hbar(3\pi^{2}n)^{1/3}}{ne^{2}\rho}$ **(10)**

which can be rearranged as:

$l_{mfp}=\frac{\hbar\sqrt[3]{3\pi^{2}}}{e^{2}\rho\sqrt[3]{3n^{2}}}$ **(11)**

The Hall carrier concentration and resistivity values obtained from Hall effect measurements were used for all calculations.

**References**

[1] (CIE), I. C. o. I. "CIE standard illuminant D65." <https://doi.org/10.25039/CIE.DS.hjfjmt59>.

[2] (CIE), I. C. o. I. *CIE 1964 colour-matching functions.* (Vienna, Austria).

[3] CIE, C. 1978. "Publication No. 15, Supplement Number 2 (E-1.3. 1, 1971): Official recommendations on uniform color spaces, color-difference equations, and metric color terms." *Commission Internationale de L’Eclairage*.

[4] Jia, J., Muto, Y., Oka, N., and Shigesato, Y. 2012. "Annealing effects on Ta doped SnO2 films." *MRS Online Proceedings Library (OPL)* 1454: 245-251.
